# BioCNTs Mediated Delivery of Systemically Mobile Small RNAs via Leaf Spray to Control Both Tomato DNA and RNA Viruses

**DOI:** 10.1002/advs.202504889

**Published:** 2025-12-21

**Authors:** Xuedong Liu, Xiaofei Liang, Zipeng Cai, Zheng Liu, Xuefeng Wang, Changyong Zhou, Mengji Cao, Sijia Liu

**Affiliations:** ^1^ National Citrus Engineering Research Center Citrus Research Institute Southwest University Chongqing China; ^2^ College of Architectural Engineering Shenzhen Polytechnic University Shenzhen China; ^3^ College of Life Sciences and Oceanography Guangdong Provincial Key Laboratory for Plant Epigenetics Shenzhen University Shenzhen Guangdong China

**Keywords:** biopesticide, carbon nanotubes, RNA interference, tomato viral diseases, tRNA‐like structure

## Abstract

Tomato viral diseases severely restrict the safe and efficient tomato production, and current control methods are lacking. Therefore, the development of green and efficacious biopesticides is imperative. The initiation of RNA interference (RNAi) by topically applied double‐stranded RNA (dsRNA) or short hairpin RNA (shRNA) has potential applications in crop protection. However, the instability and limited mobility of exogenous naked RNAi molecules severely limit their practical large‐scale application. Here, a robust method combining dsRNA and shRNA‐mediated silencing technology with single‐walled carbon nanotubes (SWCNTs)‐based nanocarriers (BioCNTs) is developed to prevent tomato mosaic virus, tomato spotted wilt virus, and tomato yellow leaf curl virus through exogenous spraying. This method is nontoxic to tomato plants and enables effective delivery of dsRNA or shRNA for long‐term silencing. By integrating shRNAs and tRNA‐like structure (TLS) with BioCNTs, we developed a highly effective biopesticide with systemic mobility. After 14 days of spraying, the control efficacy remained high in the upper unsprayed leaves. These findings indicate that BioCNTs‐mediated dsRNA/shRNA has strong inhibitory effects, and TLS enables systematic movement of shRNA in plants, maintaining long‐term virus silencing efficacy. Therefore, the simple and low‐cost BioCNTs‐TLS system holds significant potential as an effective, green, and sustainable tool for controlling plant viral diseases.

## Graphical abstract

A carbon nanotube‐based biopesticide system (BioCNTs) enables efficient delivery and systemic movement of RNAi molecules to control major tomato viruses. By integrating shRNA and tRNA‐like structures (TLS) with BioCNTs, this green and scalable approach achieves long‐term silencing and high antiviral efficacy through simple foliar spraying.

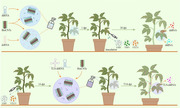

## 1 Introduction

Sustainable agriculture faces increasing challenges due to population growth, climate change, and limited arable land. Tomatoes are important economic vegetable crops worldwide, and the occurrence of viral diseases severely threatens safe tomato production. To date, at least 136 viruses have been reported to infect tomatoes worldwide, of which approximately 44 viruses are a threat to tomato production and quality in China [1, 2]. Among them, tomato spotted wilt virus (TSWV), tomato mosaic virus (ToMV), and tomato yellow leaf curl virus (TYLCV) are the main viruses that have severely threatened tomato safety production in recent years, causing up to 100% yield loss [2]. Owing to the wide host range of these three viruses, it is difficult to control their transmission vectors, and no specific chemicals are available for controlling these three viruses [3]. Therefore, the development of new biopesticides is of great scientific and practical importance for the prevention and control of tomato viral diseases.

RNA interference (RNAi) has emerged as a promising and economically viable strategy for targeted virus and host pest management [4]. A key feature of RNAi in plants is the processing of double‐stranded RNA (dsRNA) into small interfering RNA (siRNA) by DICER LIKE (DCL) enzymes. The siRNA is then incorporated into RNA‐induced silencing complexes, leading to specific degradation of any RNA that shares sequence similarity with the inducing dsRNA [4]. Some studies have reported that dsRNA or hairpin RNA (hpRNA) derived from sequences of plant virus species can protect plants from viral infection [5, 6, 7]. Therefore, RNAi‐based spraying‐induced gene silencing has shown promising results in controlling plant viruses locally and has become a novel plant protection method [8, 9]. However, for a significant RNAi response, sprayed RNAi molecules must overcome several barriers of the leaf surface prior to uptake and then stably translocate to various parts of the plant for systemic protection [10, 11].

Nanomaterials have been proven to be effective carriers for delivering nucleic acids to intact plant tissues, offering species‐independent delivery, protection against nuclease degradation, minimal damage to plant tissues and genomes, controlled cargo release, and specific organelle targeting [12, 13]. Previous studies have shown that clay nanosheets can protect dsRNA from degradation on the leaf surface to sustain virus resistance [14]. This progress reveals the potential of using nanomaterials in RNAi applications to assist crops in long‐term defense against plant viruses. Other nanomaterials, such as carbon nanotubes (CNTs), graphene oxide nanoparticles, gold nanoclusters, carbon dots, and DNA origami, have also been used for siRNA delivery into intact plants, but their effectiveness in long‐term defense against plant viruses remains to be verified. Among these materials, CNTs have shown promise for siRNA delivery and high silencing efficacy in intact plant cells [15]. Compared to other nanomaterials, single‐walled carbon nanotubes (SWCNTs) possess unique structural advantages that enable direct penetration of the plant cell wall and facilitate the transport of biomacromolecules [16]. Their high aspect ratio and nanoscale diameter (0.7–2 nm) make passive diffusion through cell wall pores (10–20 nm) theoretically feasible [15]. In addition, recent studies have proposed a lipid‐mediated mechanism known as Lipid Exchange Envelope Penetration (LEEP), in which nanoparticle surfaces transiently interact with membrane lipids to form a lipid envelope that promotes cellular entry [17]. This model has been supported by experimental evidence across various plant systems [12, 18]. Further investigations suggest that surface functionalization of SWCNTs can enhance their compatibility with plant tissues and facilitate lipid exchange, thereby improving membrane penetration efficiency [18]. However, the complex preparation method, low loading efficiency, short duration, and inability to provide systematic protection limit the application of SWCNTs. Recently, we developed a simple, fast, and instrument‐free method for preparing SWCNTs‐based nanocarriers, which has the advantages of low cost, large‐scale preparation, high nucleic acid loading efficiency, and compatibility with various plant species [19]. Here, we assess the potential of this nanocarrier for the development of novel RNAi nanobiopesticides.

Plant endogenous siRNAs, microRNAs, and mRNAs can move locally between cells through intercellular filaments or over long distances through the vascular system of the phloem [20, 21]. However, exogenous direct application of dsRNA or siRNA can only exert gene silencing effects at the spray site or over shorter distances and does not function effectively over long distances [10]. Studies have shown that some virus genomes contain conserved tRNA‐like structures (TLS) in their 3' untranslated regions (UTRs), which can be bound by plant tRNA‐binding proteins or modifying proteins, thereby mediating the intercellular or long‐distance movement of viral RNA [22]. Recently, the use of TLS sequences for long‐distance movement of nonmobile RNA in plants has been reported [23]. Moreover, when the TLS sequence is fused with Cas9 and gRNA transcripts, RNA can be transferred from transgenic rootstocks to grafted wild‐type scions, and gene‐edited plants can be obtained from transgene‐free scions [24]. Therefore, it is worth exploring whether TLS motifs can also facilitate the systemic movement of exogenously sprayed RNAi molecules in nano RNAi‐mediated antiviral biopesticides, ultimately achieving the objective of long‐term systemic control of viral tomato diseases in whole plants.

In this study, we identified sRNA fragments that are effective at preventing ToMV, TSWV, and TYLCV infection, and single‐walled carbon nanotubes (SWCNTs)‐based nanocarrier PEI‐single‐stranded DNA (ssDNA)‐SWCNTs (BioCNTs) can effectively carry dsRNA and shRNA fragments into complete plant cells. In addition, BioCNTs can effectively prolong the action time and inhibitory efficacy of dsRNA and shRNA. Then, we introduced the TLS sequence before the shRNA, confirmed that the TLS can assist in the systematic movement of exogenous shRNA, and further increased the inhibitory efficacy and persistence. These results demonstrate that our developed BioCNTs‐mediated dsRNA and shRNA delivery system can be an effective, green, and sustainable tool for the precise control of several important viral diseases in tomatoes. Furthermore, the application of TLS in RNAi technology for plant disease control offers new insights into increasing RNAi efficacy.

## 2 Results

### 2.1 Characterization of the BioCNTs‐dsRNA/shRNA Nanoparticle System

**FIGURE 1:**
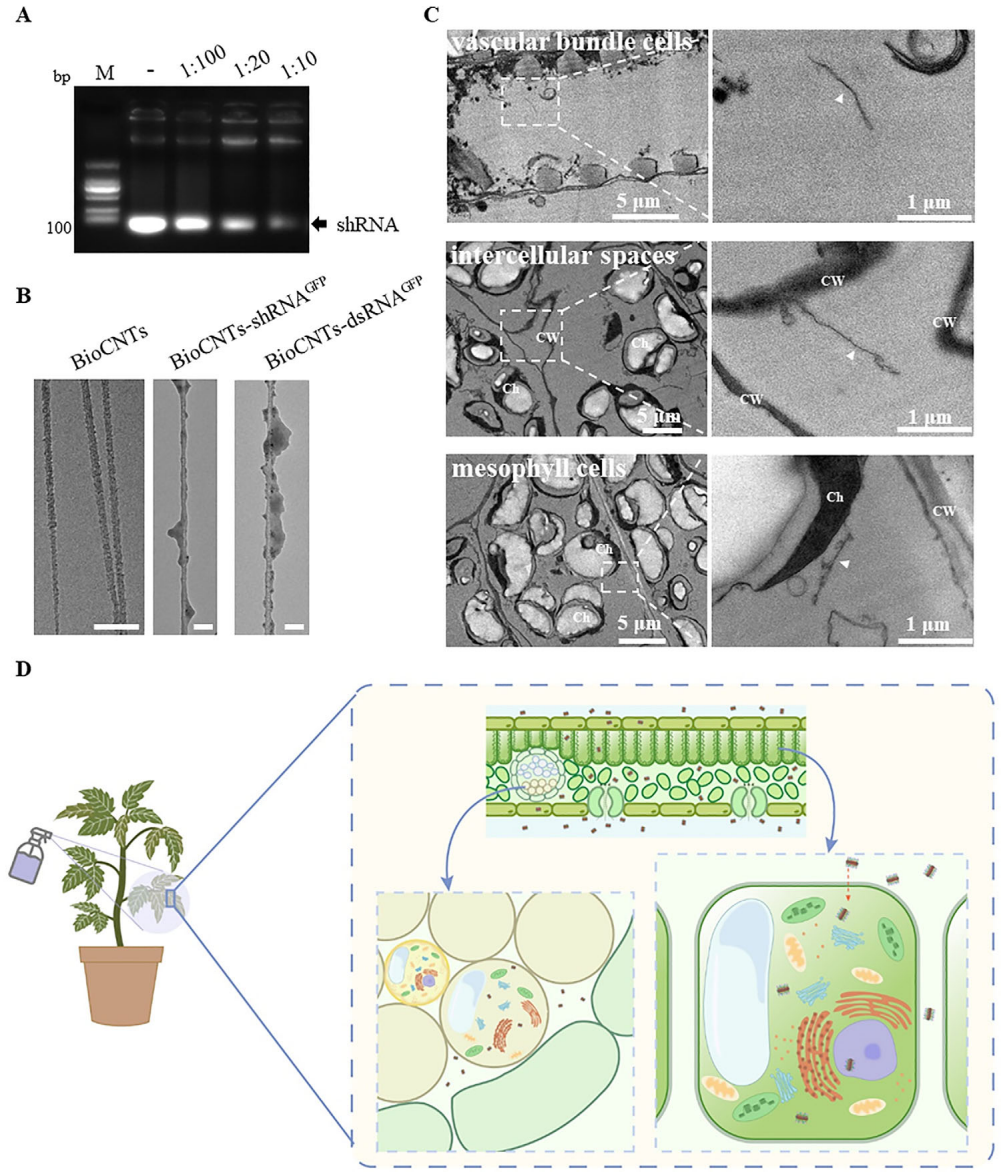
Characterization of dsRNA or shRNA loading on BioCNTs. A) Various amounts of BioCNTs were used to load 1 µg shRNA^GFP^, respectively. The agarose gel electrophoresis results showed that the optimal loading mass ratio is 1:10. B) Transmission electron microscopy (TEM) images of BioCNTs, BioCNTs‐shRNA^GFP^, and BioCNTs‐dsRNA^GFP^. C) Characterization of BioCNTs‐dsRNA entry into tomato leaf cells. Nanoparticles are observed in vascular bundle cells (the upper panel), intercellular spaces (the middle panel), and cytoplasm of mesophyll cells (the lower panel). The white arrow‐heads indicate BioCNTs. Scale bars for the full‐size image are 5 µm, and for the zoomed‐in image are 1 µm. CW, cell wall; Ch, chloroplast. D) The distribution schematic illustration of BioCNTs in tomato leaves.

We previously developed BioCNTs (PEI‐ssDNA‐SWCNTs), which can be prepared in large quantities via a simple, fast, and efficient method without the use of complex instrumentation [19]. Specifically, compared with PEI‐SWCNTs [12, 16], ssDNA‐wrapped SWCNTs allow for higher initial loading and dispersion efficiency, while the subsequent precipitation‐based purification significantly increases the final product's yield and purity (Table S1). To examine the loading efficiency of BioCNTs for RNAi molecules, we used various amounts of BioCNTs to load 1 µg shRNA^GFP^ and found that the optimal loading mass ratio was 1:10 (BioCNTs: shRNA) (Figure 1A). Then, we calculated the loading efficiency of BioCNTs on shRNA, and dsRNA, and the results showed that at a ratio of 1:10, the loading efficiency could all reach over 89% (Figure S1).

Then, we observed the binding of shRNA^GFP^ and dsRNA^GFP^ on the BioCNTs and examined the diameter of each type of BioCNTs via transmission electron microscopy (TEM). As shown in Figure 1B (the left panel), the diameter of the BioCNTs was approximately 10‐20 nm. After incubation with shRNA or dsRNA, we successfully observed the shRNA and dsRNA loaded on the BioCNTs, and the diameter at this stage was approximately 20–30 nm (Figure 1B, the middle and right panel).

Furthermore, to evaluate whether BioCNTs can protect RNA from RNase A‐mediated degradation, we performed a gel electrophoresis assay to assess the degradation of shRNA following RNase A treatment. As treatment time increased, shRNA^GFP^ incubated with RNase A showed gradually weakened bands. At 5 min post‐treatment, no visible bands remained, indicating complete degradation of free RNA by RNase A (Figure S2, left panel). In contrast, when loaded shRNA^GFP^ onto BioCNTs and treated with RNase A, the BioCNTs‐bound shRNA^GFP^ did not migrate from the well during electrophoresis and presented as fluorescence in the well. Even after 15 min of RNase A exposure, fluorescence was still detectable in the well (Figure S2, right panel), suggesting that BioCNTs effectively protect RNA from enzymatic degradation.

To determine whether BioCNTs can successfully deliver RNAi molecules into plant cells through surface spraying, we sprayed BioCNTs‐dsRNA^GFP^ on tomato leaves, and at 1 day post‐treatment (dpt), we collected leaf samples for observation via TEM. The results revealed BioCNTs‐dsRNA^GFP^ in vein vascular bundle cells, intercellular spaces, and the cytoplasm of mesophyll cells (Figure 1C). And we proposed a distribution schematic illustration of BioCNTs in tomato leaves (Figure 1D). The detection of BioCNTs within mesophyll cell cytoplasm following surface spraying implies that BioCNTs can bypass the cell wall barrier via passive diffusion, and subsequently penetrate the plasma membrane through lipid exchange envelope mechanisms previously described for nanomaterials in plants.

### 2.2 BioCNTs are Safe for Living Plants

**FIGURE 2:**
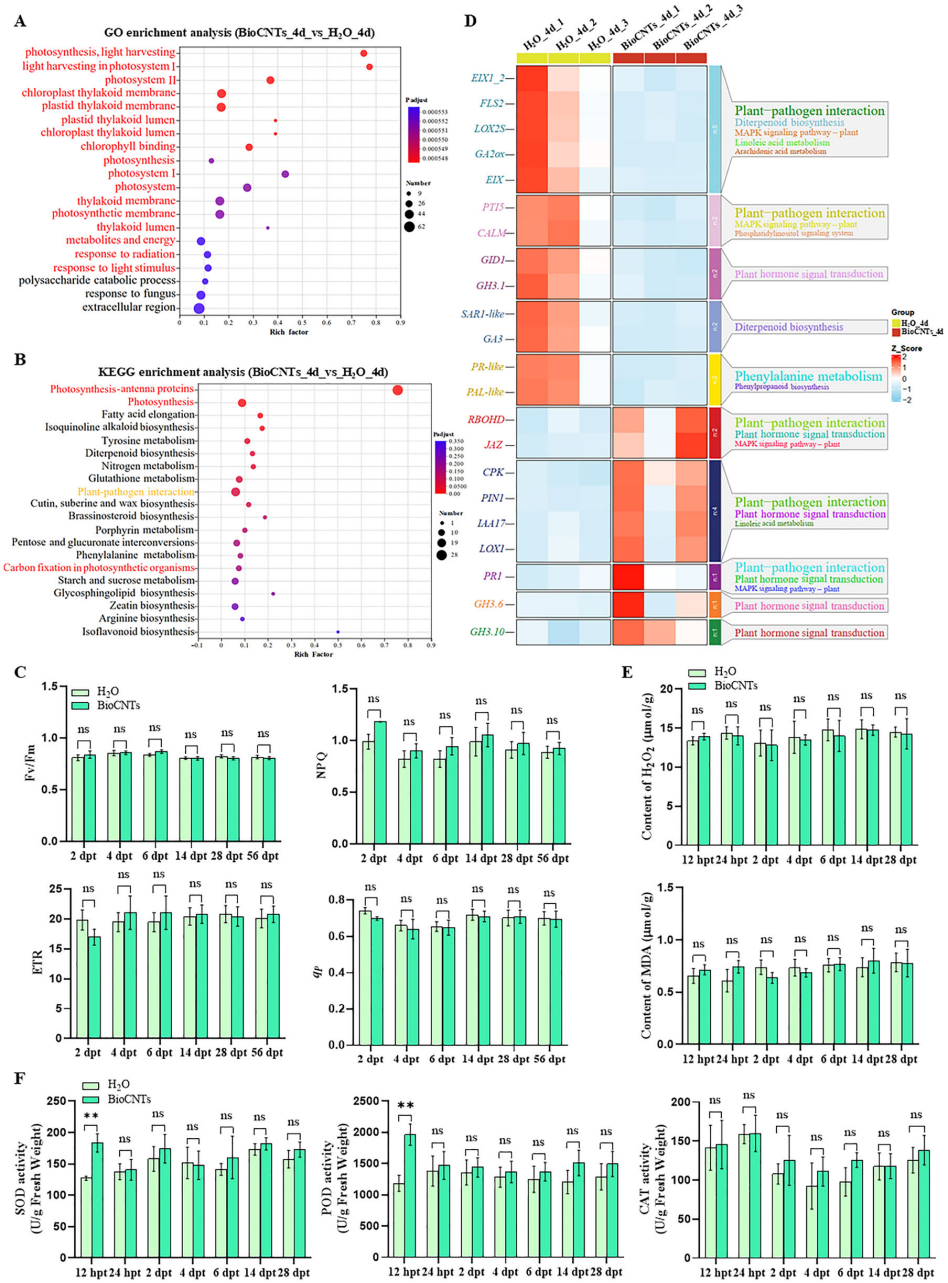
BioCNTs do not significantly alter the immune system of tomatoes. (A) The GO enrichment analysis of differentially expressed genes (DEGs) at 4 days post BioCNTs treatment (dpt). (B) The KEGG enrichment analysis of DEGs at 4 dpt. (C) Analyses of PSII efficiency (*Fv*/*Fm*), the non‐photochemical quenching (NPQ), the electron transfer rate (ETR), and the photochemical quenching coefficient (*q*_P_) in the treated leaves from the BioCNTs‐, or H_2_O‐inoculated (control) tomato plants at 2, 4, 6, 14, 28, and 56 dpt. (D) The combined analysis of clustering and pathway enrichment of the plant resistance pathway under BioCNTs treatment at 4 dpt. (E) Changes of reactive oxygen species in tomato leaves treated with BioCNTs, and H_2_O (control) at 12 hpt, 24 hpt, 2 dpt, 4 dpt, 6 dpt, 14 dpt, and 28 dpt. (F) Changes of enzyme activities of SOD, POD, and CAT in tomato leaves treated with BioCNTs, and H_2_O (control) at 12 hpt, 24 hpt, 2 dpt, 4 dpt, 6 dpt, 14 dpt, and 28 dpt. In (C,E,F), **, *p* < 0.01, ns, not significant, determined using the two‐tailed Student's *t*‐test, error bars were SEM. These experiments were performed three times and had at least six biological replicates per treatment.

Nanomaterials are crucial for the safety of living organisms [25]. To investigate whether the application of BioCNTs has cytotoxic effects on plants, we conducted Illumina RNA sequencing to investigate alterations in the transcriptomes of BioCNTs‐treated tomato plants. Leaf samples were collected from tomato seedlings at 4 days after BioCNTs treatment, and total RNA was then extracted for further analysis. The expression distribution results indicated that BioCNTs treatment did not significantly affect the total number of expressed genes (Figure S3A). Specifically, a total of 1057 differentially expressed genes (DEGs) (639 with upregulated expression and 418 with downregulated expression) were identified (Figure S3B; Table S2). Further analysis via a Gene Ontology (GO) enrichment analysis revealed that the majority of the DEGs were enriched in the photosynthesis pathway (Figure 2A). Kyoto Encyclopedia of Genes and Genomes (KEGG) enrichment analysis revealed that the DEGs were highly enriched in photosynthesis, plant‐pathogen interaction, and metabolism pathways (Figure 2B). To conduct a more detailed analysis of the metabolic pathways underlying the differences in gene expression, we generated a grouped heatmap of gene expression (Figure S4A). The top three subclusters (red, green, and blue) in the heatmap were subsequently subjected to KEGG analysis to evaluate potential key genes regulated by BioCNTs treatment. The genes in subclusters 1 (red, 215) and 3 (blue, 371) were significantly upregulated under BioCNTs treatment and were associated with photosynthesis, sucrose starch metabolism, and plant‐pathogen interaction pathways (Figure S4B, C). Moreover, genes in subcluster 2 (green, 412) were significantly downregulated under BioCNTs treatment and were associated with mainly plant‐pathogen interactions and the MAPK signaling pathway (Figure S4D).

Given that most DEGs were enriched in the photosynthesis pathway, we conducted clustering and pathway analysis on the genes involved in the photosynthesis pathway and found that, with the exceptions of *psbA* and *Chloroa_b‐bind*, all other photosynthesis‐related genes were upregulated at 4 days post BioCNTs treatment (Figure S5). The main function of chloroplasts is photosynthesis. Therefore, to investigate the effect of BioCNTs spraying on plant photosynthesis, we measured the photosynthesis of tomato plants at 2, 4, and 6 days after BioCNTs spraying. These results indicate that the spraying of BioCNTs does not significantly affect the photosynthesis of plants (Figure 2C).

Considering that BioCNTs would likely be applied continuously over an extended period in agricultural production, we treated tomato plants with BioCNTs via foliar spraying three times during the growth period. Then, we measured the photosynthesis at 14, 28, and 56 dpt, the results showed that the spraying of BioCNTs did not significantly affect the photosynthesis of plants (Figure 2C). Moreover, we analyzed the DEGs in the photosynthesis pathway at 14 and 28 dpt (Table S3‐S9). The results showed that most photosynthesis‐related genes were not significantly changed under BioCNTs treatment at 14 dpt and 28 dpt (Figure S6).

Furthermore, we examined changes in genes associated with plant resistance pathways at 4, 14, and 28 dpt (Table S10). Combined clustering and pathway enrichment analyses revealed complementary expression patterns among homologous genes (Figure 2D; Figure S7). In addition, under stress, plant cells rapidly accumulate reactive oxygen species (ROS). However, only the expression of *RBOHD* was significantly induced by BioCNTs at 4 dpt (Figure 2D). To verify whether BioCNTs treatment could induce a defense response, we assessed changes in ROS accumulation and enzyme activities in BioCNTs‐treated and H_2_O‐treated (control) tomato leaves. The results revealed that there was no significant change in the levels of malondialdehyde (MDA) or H_2_O_2_ compared with the control (Figure 2E). The enzyme activities of superoxide dismutase (SOD) and peroxidase (POD) were significantly up‐regulated at 12 h post BioCNTs treatment (hpt), whereas at 24 hpt, the enzyme activities of SOD, POD, and catalase (CAT) were not significantly different from those in the H_2_O‐treated control leaves (Figure 2F). These data revealed that BioCNTs treatment increased the expression of the photosynthesis pathway and several redox‐related genes at the early treatment stage, but did not significantly affect photosynthesis or intracellular ROS levels, indicating that BioCNTs does not trigger a strong immune response or exert substantial negative impacts on the healthy growth of tomatoes.

### 2.3 BioCNTs Increased the Inhibitory Efficacy of RNAi Molecules against Viral Diseases

**FIGURE 3:**
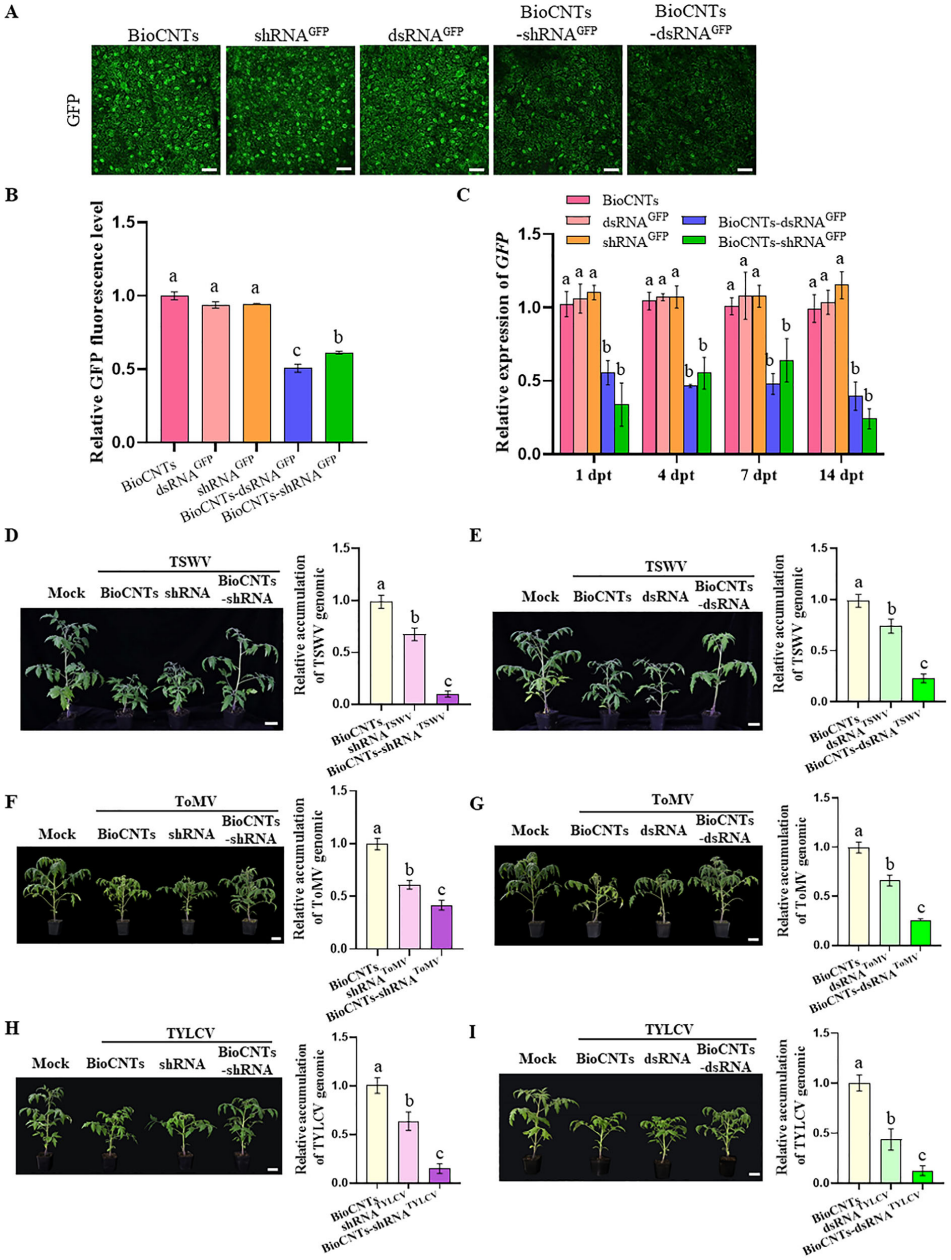
BioCNTs enhanced the prevention efficiency of dsRNA/shRNA against viral diseases. (A) At 4 dpt, treated‐leaves of 16C *N. benthamiana* were visualized by a Leica confocal microscope. The BioCNTs‐treated leaves were used as a control. Bars = 50 µm. (B) GFP fluorescence intensity statistics. The mean number and the SEM for each treatment are from n = 6 leaves from three independent biological replicates. The fluorescence intensity was determined using the ImageJ software. (C) The RT‐qPCR result showed that the expression levels of *GFP* in the BioCNTs‐dsRNA^GFP^ and BioCNTs‐shRNA^GFP^‐treated plants were all indeed down‐regulated significantly. (D,E) The BioCNTs‐shRNA^TSWV^ and BioCNTs‐dsRNA^TSWV^‐treatment inhibited TSWV infection with milder symptoms, and RT‐qPCR results showed significantly decreased TSWV RNA accumulation in tomato plants (BioCNTs‐shRNA^TSWV^ / BioCNTs‐dsRNA^TSWV^) compared with that in the control (BioCNTs, shRNA^TSWV^, and dsRNA^TSWV^‐treatment) plants. (F,G) The BioCNTs‐shRNA^ToMV^ and BioCNTs‐dsRNA^ToMV^‐treatment inhibited ToMV infection with milder symptoms, and RT‐qPCR results showed significantly decreased ToMV RNA accumulation in tomato plants (BioCNTs‐shRNA^ToMV^ / BioCNTs‐dsRNA^ToMV^) compared with that in the control (BioCNTs, shRNA^ToMV^, and dsRNA^ToMV^‐treatment) plants. (H,I) The BioCNTs‐shRNA^TYLCV^ and BioCNTs‐dsRNA^TYLCV^‐treatment inhibited TYLCV infection with milder symptoms, and RT‐qPCR results showed significantly decreased TYLCV DNA accumulation in tomato plants (BioCNTs‐shRNA^TYLCV^ / BioCNTs‐dsRNA^TYLCV^) compared with that in the control (BioCNTs, shRNA^TYLCV^, and dsRNA^TYLCV^‐treatment) plants. Scale bars = 5 cm (D–I). The different letters above each bar in B‐I indicated statistically significant differences as determined by a one‐way ANOVA followed by Tukey's multiple test (*p* < 0.05), error bars were SEM. These experiments were performed three times and had at least six biological replicates per treatment.

To investigate the function of BioCNTs in dsRNA/shRNA‐mediated RNA silencing, we used GFP‐transgenic 16C *Nicotiana benthamiana* plants to test the GFP expression level. The shRNA^GFP^, dsRNA^GFP^, BioCNTs‐shRNA^GFP^, or BioCNTs‐dsRNA^GFP^ (approximately 4 µg of shRNA or dsRNA and 0.4 µg of BioCNTs per leaf) was sprayed on the leaves of 16C *N. benthamiana*, respectively. The BioCNTs‐treated plants were used as a control. At 7 dpt, confocal microscopy results indicated that the fluorescence intensity of the BioCNTs‐shRNA^GFP^/dsRNA^GFP^‐treated leaves was significantly lower than that of the BioCNTs and shRNA^GFP^/dsRNA^GFP^‐treated leaves (Figure 3A). The fluorescence statistics are consistent with the fluorescence observation results (Figure 3B). The RT‐qPCR results revealed that, compared with the BioCNTs, naked‐dsRNA^GFP^ or shRNA^GFP^, the BioCNTs loading strategy (BioCNTs‐dsRNA^GFP^/shRNA^GFP^) can maintain approximately 50%–60% GFP silencing efficacy for 14 days (Figure 3C). These results indicated that BioCNTs can effectively deliver dsRNA and shRNA into plant cells via foliar spraying, facilitating gene silencing induced by exogenous dsRNA and shRNA. Notably, shRNA achieves comparable silencing efficacy to dsRNA.

Furthermore, we tested the efficacy of local spraying of BioCNTs‐dsRNA, BioCNTs‐shRNA, or BioCNTs compared with naked‐dsRNA and shRNA in providing RNAi‐mediated protective effects against ToMV, TSWV, or TYLCV in tomato. We performed sequence alignment on the ToMV‐encoded CP (Figure S8A,B), the TSWV‐encoded CP (Figure S9A,B), and the TYLCV‐encoded AC4 (Figure S10A,B) and selected the conserved 21 nt sequence as the target shRNA and dsRNA sequence (Figures S8C, S9C, and S10C). BLASTn analysis revealed that the selected 21 nt sequence is specific and that there are no nonspecific targets. The target shRNA and dsRNA were subsequently expressed and purified in HT115 (DE3), an RNase III‐deficient *Escherichia coli* (Figure S11A,B).

A loading ratio of 1:10 BioCNTs‐dsRNA/shRNA (approximately 4 µg of shRNA or dsRNA and 0.4 µg of BioCNTs per leaf) was used in the tomato protection experiments. The four‐leaf‐stage tomato plants were first treated with BioCNTs‐dsRNA^ToMV/TSWV/TYLCV^/shRNA^ToMV/TSWV/TYLCV^, and at 1 dpt, the treated leaves were inoculated with ToMV, TSWV, or TYLCV. Then, we determined the infection efficacy of these three viruses at 30 days post‐inoculation (dpi) via RT‐PCR (Figure S12). Furthermore, we determined the accumulation levels of three viruses in successfully infected tomato plants via RT‐qPCR. Compared with BioCNTs treatment, both naked shRNA and dsRNA resulted in an approximately 30%–40% reduction in virus accumulation (Figure 3D–I, right panel). However, these treatments did not alleviate the symptoms caused by the virus (Figure 3D–I, left panel). In contrast, there were clearly fewer symptoms associated with these viruses in the BioCNTs‐shRNA or BioCNTs‐dsRNA‐treated tomato plants than in the BioCNTs control plants (Figure 3D–I, left panel). Compared with the BioCNTs control, both BioCNTs‐shRNA and BioCNTs‐dsRNA application significantly inhibited virus accumulation, with approximately 88% and 76% reductions in TSWV, 58% and 74% reductions in ToMV, and 85% and 87% reductions in TYLCV, respectively (Figure 3D–I, right panel).

To verify the robustness of this system, we designed shRNA constructs targeting the replication‐related gene of ToMV and TSWV (shRNA^Rep^, Figures S8D and S9D), and the AV1 gene of TYLCV (shRNA^AV1^, Figure S10D), and then tested their antiviral efficacy against all three viruses. The results showed that when shRNA targets the replication‐related gene of ToMV, TSWV, or the AV1 gene of TYLCV, BioCNTs‐shRNA treatment can also effectively weaken the symptoms of the three viruses, respectively, and significantly reduce the level of virus genome accumulation (Figure S13).

Then, we performed resistance tests on two other tomato cultivars (Moneymaker and MicroTom) to determine the resistance differences across varieties. We sprayed the BioCNTs, shRNA^ToMV/TSWV/TYLCV^, and BioCNTs‐shRNA^ToMV/TSWV/TYLCV^ on these two tomato varieties, respectively, and then inoculated TSWV, ToMV, or TYLCV at 1 dpt. Consistent with the results of AC tomato cultivar, the virus symptoms of the BioCNTs‐shRNA^ToMV/TSWV/TYLCV^ treatment group were significantly reduced, and the level of virus accumulation was also significantly lower than that of the BioCNTs, and shRNA treatment groups (Figure S14).

Given that field conditions often involved mixed infections, we conducted co‐infection experiments to evaluate the synergistic control efficacy of BioCNTs. We sprayed the BioCNTs, shRNA^To+TY^, or BioCNTs‐shRNA^To+TY^ on the AC tomato cultivar, respectively. At 1dpt, plants were inoculated with TYLCV, followed by ToMV inoculation at 14 days after TYLCV infection. At 30 days post TYLCV inoculation, sprayed leaves were collected for further analysis. The results showed that BioCNTs‐shRNA^To+TY^ treatment significantly alleviated the virus symptoms and markedly reduced the accumulation levels of both ToMV and TYLCV (Figure S15). These findings indicated that our system confers broad‐spectrum antiviral protection across different tomato cultivars. The above results imply that BioCNTs facilitate the functional delivery of dsRNA/shRNA, supporting their use in virus control.

### 2.4 The TLS Can Assist in the Systematic Movement of Exogenous shRNA in Plants

**FIGURE 4:**
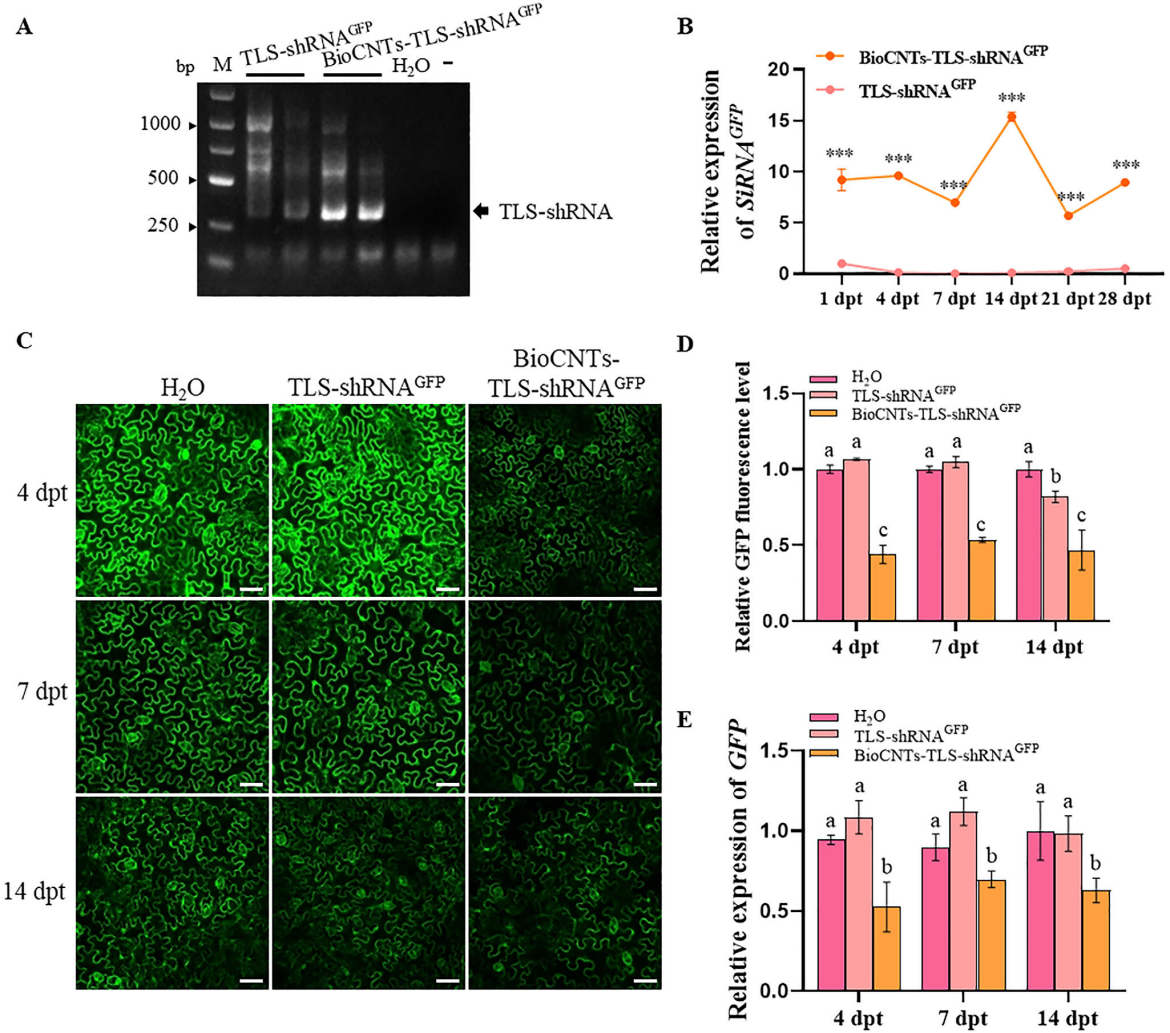
The TLS can assist in the systematic movement of exogenous shRNA in plants. (A) The TLS‐shRNA^GFP^ was tested in the upper untreated‐leaves in TLS‐shRNA^GFP^ and BioCNTs‐TLS‐shRNA^GFP^‐treated tomato plants. M, Marker. (B) The stem‐loop RT‐qPCR determined the accumulation level of siRNA^GFP^ in the upper leaves at 1, 4, 7, 14, 21, and 28 dpt. The relative expression level of siRNA^GFP^ in TLS‐shRNA^GFP^‐treatment plants at 1 dpt was taken to be 1.00. Asterisks indicate the statistical differences between treatments (***, *p* < 0.001), determined using the two‐tailed Student's *t*‐test, error bars were SEM. (C) At 4, 7, and 14 dpt, the upper untreated‐leaves of 16C *N. benthamiana* were visualized by a Leica confocal microscope. The H_2_O‐treated leaves were used as a control. Bars = 50 µm. (D) The GFP fluorescence intensity statistics. The fluorescence intensity was determined using the ImageJ software. The fluorescence intensity in H_2_O‐treated leaves in each time point was taken to be 1.00, respectively. (E) The RT‐qPCR assay determined the relative expression level of *GFP* in 16C *N. benthamiana* at 4, 7, and 14 dpt. The different letters above each bar in (D,E) indicated statistically significant differences as determined by a one‐way ANOVA followed by Tukey's multiple test (*p* < 0.05). The mean number and the SEM for each treatment are from n = 6 leaves from three independent biological replicates.

Given that tRNA‐like structures (TLS) mimic canonical tRNA and mediate long‐distance RNA transport in plants [22], we hypothesized that fusing TLS to shRNA would enable enhanced systemic silencing. First, we calculated the loading efficiency of BioCNTs on TLS‐shRNA, and the results showed that at a ratio of 1:10, the loading efficiency reached 89.07% (Figure S16). To verify whether TLS can mediate the systemic trafficking of exogenous shRNAs, we sprayed BioCNTs, TLS‐shRNA^GFP^, and BioCNTs‐TLS‐shRNA^GFP^ on the second true leaf of wide‐type *N. benthamiana*. The presence of TLS‐shRNA^GFP^ was detected in the fourth true leaf from the upper unsprayed leaves at 4 dpt (Figure 4A). At 1, 4, 7, 14, 21, and 28 dpt, we collected the fourth true leaf from the upper part that had not been sprayed, and tested the accumulation of siRNA‐GFP via stem‐loop RT‐qPCR assay. The results revealed that siRNA‐GFP was detected in the upper systemic leaf after spraying, and the accumulation level was highest at 14 dpt (Figure 4B). We then sprayed BioCNTs‐TLS‐shRNA^GFP^ on 16C *N. benthamiana* to further observe the effect of TLS‐shRNA^GFP^ in regulating *GFP* expression. At 4, 7, and 14 dpt, the upper untreated leaves were collected for confocal microscopy analysis, and the results revealed that the GFP fluorescence intensity significantly decreased in the BioCNTs‐TLS‐shRNA^GFP^‐treated upper leaves (Figure 4C,D). We subsequently performed an RT‐qPCR assay to confirm the *GFP* mRNA transcript level. Similar to the confocal microscopy results, we found that the mRNA transcript levels of *GFP* were significantly reduced in the BioCNTs‐TLS‐shRNA^GFP^‐treated plants compared with BioCNTs‐treated plants (Figure 4E). These results suggest that TLS can assist in the long‐term systemic movement of exogenous shRNAs in plants via BioCNTs delivery systems.

### 2.5 BioCNTs Can Increase the Ability and Stability of TLS‐shRNAs for Virus Prevention

**FIGURE 5:**
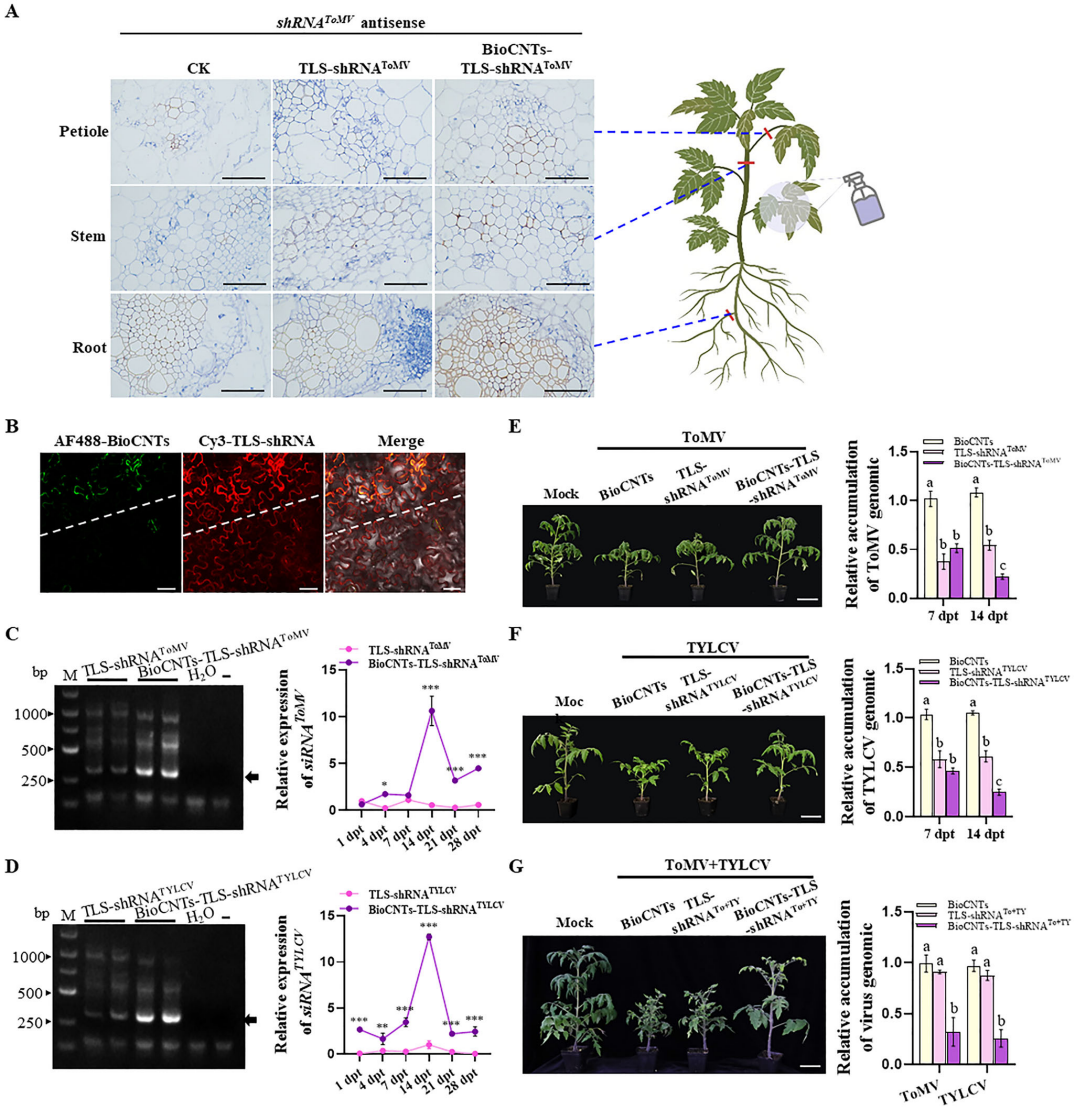
BioCNTs can enhance the ability and stability of TLS‐shRNA in virus prevention. (A) RNA in situ hybridization with DIG‐labeled probes to shRNA^ToMV^ of the roots, stems, and petioles tissues of the upper untreated leaf from BioCNTs, TLS‐shRNA^ToMV^, and BioCNTs‐TLS‐shRNA^ToMV^‐treated tomato plants. Scale bars = 15 µm. (B) Confocal assay was used to determine the mobility of BioCNTs and TLS‐shRNA in plant cells. Bars = 50 µm. (C,D) The left panel showed the TLS‐shRNA^ToMV^ and TLS‐shRNA^TYLCV^ were tested in the upper untreated‐leaves in tomato plants. M, Marker. The right panel showed that the stem‐loop RT‐qPCR determined the accumulation level of siRNA^ToMV^ and siRNA^TYLCV^ in the upper leaves at 1, 4, 7, 14, 21, and 28 dpt. The relative expression level of siRNA^ToMV/TYLCV^ in TLS‐shRNA^ToMV/TYLCV^‐treatment plants at 1 dpt was taken to be 1.00. Asterisks in (C,D) indicate the statistical differences between treatments (*, *p* < 0.05; **, *p* < 0.01; ***, *p* < 0.001), determined using the two‐tailed Student's *t*‐test, error bars were SEM. E) The BioCNTs‐TLS‐shRNA^ToMV^‐treatment inhibited ToMV infection with milder symptoms. The RT‐qPCR results showed the ToMV RNA accumulation in TLS‐shRNA^ToMV^ and BioCNTs‐TLS‐shRNA^ToMV^‐treated tomato plants at 7 dpt and 14 dpt. (F) The BioCNTs‐TLS‐shRNA^TYLCV^‐treatment inhibited TYLCV infection with milder symptoms. The RT‐qPCR results showed the TYLCV RNA accumulation in TLS‐shRNA^TYLCV^ and BioCNTs‐TLS‐shRNA^TYLCV^‐treated tomato plants at 7 dpt and 14 dpt. (G) BioCNTs‐TLS‐shRNA^To+TY^‐treatment inhibited both ToMV and TYLCV infection with milder symptoms. The RT‐qPCR results showed the ToMV RNA and TYLCV DNA accumulation in TLS‐shRNA^To+TY^ and BioCNTs‐TLS‐shRNA^To+TY^‐treated tomato plants at 14 dpt. Scale Bars = 5 cm (E–G). The different letters above each bar in (E–G) indicated statistically significant differences as determined by a one‐way ANOVA followed by Tukey's multiple test (*p* < 0.05), error bars were SEM. These experiments were performed three times and had at least six biological replicates per treatment.

To further investigate the role of TLS in long‐distance tomato virus prevention and control, we first clarified whether virus‐related shRNAs carrying TLS sequences can move from sprayed leaves to upper unsprayed leaves in treated tomato plants. The TLS sequence was connected to the N‐terminus of shRNA^ToMV^ to obtain TLS‐shRNA^ToMV^. Then, TLS‐shRNA^ToMV^ was loaded onto the BioCNTs to obtain BioCNTs‐TLS‐shRNA^ToMV^. We sprayed BioCNTs‐TLS shRNA^ToMV^ on the first true leaf of tomato, in which BioCNTs and TLS‐shRNA^ToMV^ were used as control. At 14 dpt, the roots, stems, and petioles of upper untreated leaves were collected, and the distribution of shRNA^ToMV^ and TLS were detected by in situ hybridization using *shRNA^ToMV^* antisense and *TLS* antisense probe, respectively. The results showed that both shRNA^ToMV^ and TLS were detected in roots, stems, and petioles of plants treated with BioCNTs‐TLS‐shRNA^ToMV^, while no staining was observed in BioCNTs and TLS‐shRNA^ToMV^‐treated tomato plants (Figure 5A; Figure S17). To further confirm the systemic mobility of TLS‐shRNA, we labeled BioCNTs with Alexa Fluor 488 and TLS‐shRNA with Cy3. Following foliar application, fluorescence imaging revealed Cy3 signals in adjacent tissue regions where Alexa Fluor 488 fluorescence was absent, indicating its intrinsic mobility independent of the BioCNTs carrier (Figure 5B, below the dashed line). These findings support the role of TLS in facilitating systemic translocation of the RNAi construct.

Considering that TLS could assist in the systematic movement of exogenous shRNA, we also analyzed the DEGs in photosynthesis‐related pathway and plant resistance pathway under TLS‐shRNA^GFP^ and BioCNTs‐TLS‐shRNA^GFP^ treatment. The results showed that most of the photosynthesis‐related genes were not significantly changed at 14 dpt under TLS‐shRNA^GFP^ or BioCNTs‐TLS‐shRNA^GFP^ treatment compared with the H_2_O‐treatment control. While at 28 dpt, some photosynthesis‐related genes (*psbA, ChlH, Rubrerythrin*) were significantly up‐regulated under TLS‐shRNA^GFP^‐treatment, most of the photosynthesis‐related genes were also not significantly changed under TLS‐shRNA^GFP^ or BioCNTs‐TLS‐shRNA^GFP^ treatment (Figure S6). Moreover, most of the plant resistance pathways genes were also had no significantly changed at 14 dpt and 28 dpt in TLS‐shRNA^GFP^ and BioCNTs‐TLS‐shRNA^GFP^ treated tomato plants (Figure S7).

Then, a RNA virus (ToMV) and a DNA virus (TYLCV) were selected to determine the accumulation of TLS‐shRNA in the systemic leaves. The BioCNTs‐TLS‐shRNA ^ToMV/TYLCV^ was sprayed on the second true leaf of tomato plants, respectively. The TLS‐shRNA^ToMV/TYLCV^ was detected in the fourth upper unsprayed leaves at 4 dpt (Figure 5C,D, left panel). In addition, we analyzed the accumulation level of siRNA‐ToMV/TYLCV in the fourth upper unsprayed leaves at 1, 4, 7, 14, 21, and 28 dpt via stem‐loop RT‐qPCR, where the accumulation of both exogenous virus siRNAs was the highest at 14 dpt and then gradually decreased until 28 days (Figure 5C,D, right panel).

For the determination of whether TLS‐shRNA could inhibit virus accumulation in the upper unsprayed leaves, tomato seedlings were preinoculated with ToMV or TYLCV, and at 14 dpi, BioCNTs, TLS‐shRNA^ToMV/TYLCV^, and BioCNTs‐TLS‐shRNA^ToMV/TYLCV^ were sprayed, respectively. Compared to the TLS‐shRNA^ToMV/TYLCV^ and BioCNTs control‐treated plants, the BioCNTs‐TLS‐shRNA^ToMV/TYLCV^‐treated tomato plants had reduced symptoms of both ToMV and TYLCV infection (Figure 5E,F, the left panel). At 7 and 14 dpt, we collected the third and fourth systemic leaves from the upper unsprayed leaves for RT‐qPCR analysis. Surprisingly, compared with the BioCNTs control treatment, both naked‐TLS‐shRNA^ToMV^ and TLS‐shRNA^TYLCV^ effectively reduced the accumulation of the corresponding virus by approximately 45%–55%, which was similar to that in the BioCNTs‐TLS‐shRNA^ToMV/TYLCV^ treatment group at 7 dpt (Figure 5E,F, the right panel). However, 14 days after spraying, neither naked TLS‐shRNA^ToMV^ nor TLS‐shRNA^TYLCV^ further reduced the accumulation of the two viruses, whereas compared with the control treatment, BioCNTs‐TLS‐shRNA^ToMV/TYLCV^ reduced the accumulation of ToMV or TYLCV to approximately 80% (Figure 5E,F, the right panel). Then, we also tested the function of TLS‐shRNA^Rep/AV1^ in controlling ToMV or TYLCV infection. The results showed that BioCNTs‐TLS‐shRNA^To‐Rep^ and BioCNTs‐TLS‐shRNA^TY‐AV1^ treatments significantly alleviated viral symptoms and reduced viral accumulation levels (Figure S18). Moreover, in Moneymaker and MicroTom cultivar, BioCNT‐TLS‐shRNA^ToMV/TYLCV^ can also effectively control ToMV and TYLCV infection (Figure S19).

Then, we evaluated the antiviral efficacy of TLS‐shRNA^To+TY^ under co‐infection conditions. Compared with TLS‐shRNA^To+TY^ and BioCNTs controls, BioCNTs‐TLS‐shRNA^To+TY^‐treated tomato plants showed markedly reduced symptoms of both viruses (Figure 5G, the left panel). At 14 dpt, RT‐qPCR analysis of the third untreated systemic leaves revealed ∼75% reduction in ToMV and TYLCV accumulation (Figure 5G, the right panel).

### 2.6 BioCNTs Application Caused No Detectable Fruit Quality Decline and Mammalian Toxicological Responses

To further consider the residual effects of BioCNTs‐TLS constructs, we assessed the potential for accumulation in tomato fruit and biosafety of BioCNTs and BioCNTs‐TLS‐shRNA^GFP^. First, we sprayed the BioCNTs or BioCNTs‐TLS‐shRNA^GFP^ on tomato leaves three times during the growth period. At 28 dpt, transmission electron microscopy was used to examine the petiole where the sprayed leaves were located, the upper stem, and carpopodium. Notably, we did not detect translocation of BioCNTs from leaves to carpopodium, suggesting limited systemic movement of BioCNTs toward edible organs (Figure S20A). Upon fruits maturation, we measured fresh weight, lycopene concentration, and degrees Brix. No significant differences were observed between treated and control (H_2_O) plants (Figure S20B–E), suggesting that repeated BioCNTs application does not adversely affect tomato fruit yield or quality under the tested conditions.

To evaluate the mammalian toxicity of BioCNTs, mice were orally administered saline control, tomato fruits from untreated plants, tomato fruits from BioCNTs‐treated plants, and BioCNTs solution (0.4 mg/L). No significant differences in body weight were observed across groups (Figure S21A). Notably, histological analysis of heart, liver, spleen, lung, and kidney tissues revealed no pathological alterations in the BioCNTs group (Figure S21B). In addition, among hematological parameters, such as HGB (hemoglobin), WBC (white blood cell), RBC (red blood cell), PLT (platelet), HCT (hematocrit), no significant differences were observed between treatment groups and the saline control, indicating no evidence of hematopoietic toxicity or trigger systemic inflammation (Figure S21C).

Taken together, our data support the biocompatibility of the 0.4 mg/L BioCNTs solution (the working concentration in this study) and BioCNTs‐treated tomato plants in mice, with no evidence of systemic toxicity. These results indicate that BioCNTs‐TLS‐shRNA can provide long‐term systemic protection against viral tomato diseases, which will provide new insights for the development of RNAi technology for biopesticides.

## 3 Discussion

**FIGURE 6:**
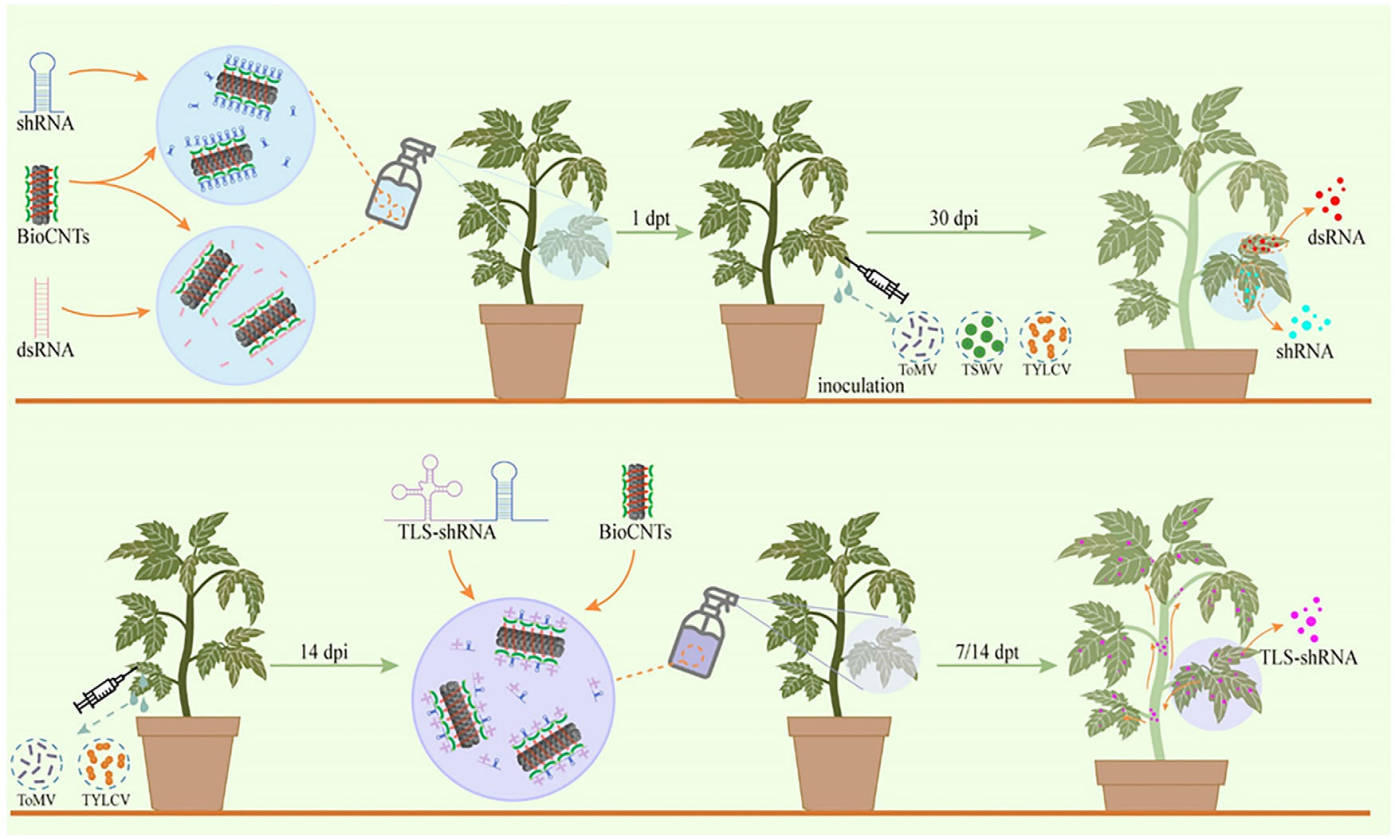
A proposed model for BioCNTs in the prevention and control of tomato virus. The upper panel showed that the BioCNTs loaded with shRNA or dsRNA were sprayed onto the surface of tomato leaves. At 1 dpt, the sprayed‐leaves were inoculated with ToMV, TSWV, or TYLCV. At 30 dpi, the sprayed leaves were collected for further detection. And the accumulation of ToMV, TSWV, and TYLCV was all significantly decreased. The lower panel showed that the tomato plants were inoculated with ToMV or TYLCV. At 14 dpi, the BioCNTs loaded with TLS‐shRNA were sprayed onto the upper first system leaf. At 7 and 14 dpt, the upper un‐sprayed leaves were collected for further detection. And the accumulation of ToMV and TYLCV were all significantly decreased on the upper un‐sprayed system leaves.

Owing to the high cost and lengthy process of genetic transformation in plants, as well as the presence of genetic transformation bottlenecks in many crops, nontransgenic exogenous methods have broader potential for application [26, 27]. The application of RNAi technology involving exogenous dsRNAs, siRNAs, or shRNAs to promote plant resistance to viruses, nematodes, pests, and fungi has been extensively studied [28, 29, 30, 31]. This study used a BioCNTs‐based nanodelivery system that effectively delivers dsRNA and shRNA into plant cells via a simple spraying method. Through the addition of a TLS, which is a system of mobile elements, exogenous RNAi molecules can be transported over long distances within the plant system (Figure 6). We can effectively control the occurrence and accumulation of RNA and DNA viral diseases in tomatoes by designing specific dsRNA or shRNA sequences for different viruses. In addition, the method of preparing BioCNTs‐based nanocarriers in large quantities through a simple process without the need for complex equipment is highly suitable for RNAi technology development for crop protection.

Currently, dsRNA is the primary RNAi molecule studied in the context of RNAi‐mediated antiviral gene silencing. For example, exogenous application of dsRNA can induce plant resistance to various viruses and prevent viral diseases, such as pepper mild mottle virus (PMMoV), zucchini yellow mosaic virus, tobacco mosaic virus (TMV), cucumber mosaic virus (CMV), alfalfa mosaic virus, and tobacco etch virus (TEV) [14, 32, 33, 34, 35]. Compared with dsRNA, shRNA can form a double‐stranded structure through complementary base pairing at both ends, which protects its ends from degradation by nucleases, increasing its stability [36]. In addition, shRNA is more specific than dsRNA and thus causes minimal nonspecific gene silencing. In this study, we downloaded the currently reported dominant virus races in the field and obtained conserved segments through sequence alignment. We then designed dsRNA and shRNA sequences from the conserved segments to address the issues of natural virus mutation and virus coinfection in the field. Our results demonstrated that exogenous application of shRNA, which designed from different targets, can effectively prevent the infection of tomato RNA and DNA viruses, and this reduction in virus accumulation is similar to that of dsRNA. Moreover, the shRNAs also showed similar disease resistance effects in three tomato varieties, indicating the generality and robustness of this BioCNTs system. The method for inducing shRNA in vitro is similar to that for inducing dsRNA. The target plasmid can be obtained via the three‐primer annealing method with a simple operation [37]. In addition, BioCNTs‐based nanocarriers display high RNA delivery efficacy with a BioCNTs/RNA weight ratio of 1:10, and can deliver multiple shRNAs into a single cell simultaneously to target different regions of the virus genome, reducing the emergence of viral escape mutants.

In recent years, a variety of nanocarriers have been developed for delivering siRNA in plants to enable RNAi‐based antiviral protection [14, 15, 28, 38]. These studies have demonstrated substantial progress in material design, delivery efficiency, and application strategies (Table S11). For instance, clay nanosheets and carbon nanocarriers are well‐suited for foliar spray applications; the former has been used to control CWV and PMMoV viruses in tobacco [14], while the latter has achieved effective silencing of the *GFP* gene in transgenic *mGFP5 Nicotiana benthamiana* [15]. DNA nanostructures exhibit high intracellular silencing efficiency and have been shown to suppress *GFP* expression in transgenic plants [38]. The TDN@CPP@PDA‐MSN system, through multifunctional surface modifications, achieved a certain degree of systemic movement and was applied to control TMV and PVY viruses in *N. benthamiana* [28]. Despite these advances, current RNA delivery systems still face challenges in RNA loading capacity, systemic silencing capacity, preparation simplicity, and cost‐effectiveness. The BioCNTs system developed in this study demonstrates integrated advantages across multiple key parameters. It can be fabricated using standard laboratory equipment, offering scalability and low production cost suitable for large‐scale deployment. Compared to other nanomaterials, BioCNTs exhibit a high loading efficiency for shRNA and dsRNA (Table S11). Notably, the incorporation of a TLS fusion enables systemic movement of RNA molecules beyond the treated leaves, overcoming the spatial limitations of locally acting nanocarriers. In tomato, BioCNTs effectively suppress multiple plant viruses, including TSWV, ToMV, and TYLCV, with siRNA detectable on leaf surfaces for up to 28 days, indicating prolonged protection. In addition, our recent findings demonstrate that BioCNTs can simultaneously deliver multiple DNA plasmids into plant cells and support their successful expression [19]. Here, BioCNTs simultaneously delivered two distinct RNAi molecules, effectively suppressing co‐infection by ToMV and TYLCV. These results highlight the potential of BioCNTs as a versatile platform for developing multiplexed antiviral strategies in other economically important crops.

Given the increasing accumulation of CNTs in the environment, nanomaterials are crucial for the safety of living organisms [25, 39, 40]. Currently, studies have revealed a positive correlation between the concentration of CNTs and their toxicity to plants [40, 41]. For SWCNTs, at low concentrations (10 mg/L), there was no significant harmful effect on pea leaves, but at high concentrations (300 mg/L), abundant epicuticular wax was generated on both leaf surfaces, and photosynthesis was impaired [41]. Studies on the toxicity of SWCNTs in mammals have shown that water‐soluble and appropriately functionalized carbon nanotubes are non‐toxic [42, 43]. In this study, we used ssDNA to disperse SWCNTs, leading to a loading mass ratio of BioCNTs: shRNA/dsRNA = 1:10, which substantially increased the transformation efficacy. The actual working concentration of BioCNTs was 0.4 mg/L, therefore avoiding damage to plants caused by high concentrations of SWCNTs. Moreover, our transcriptomic data and physiological measurements indicate that the working concentration of BioCNTs used here does not trigger strong immune responses in tomato plants and has little to no negative impact on healthy tomato growth. In addition, Star and colleagues reported that in the presence of low concentrations of H_2_O_2_, the oxidative activity of horseradish peroxidase was catalyzed by natural enzymes for carboxylation and biodegradation of SWCNTs. Within ten days, almost all nanotubes degraded. Moreover, peroxidases such as myeloperoxidase (MPO) and eosinophil peroxidase (EPO) have been shown to degrade SWCNTs [44]. Through measuring the oxidation levels of plants after spraying BioCNTs, although the levels of H_2_O_2_ and MDA did not significantly increase, the enzyme activities of SOD and POD increased 12 h after spraying. In plants, the SOD and POD play important roles in the clearance of reactive oxygen species, among which POD, MPO, and EPO belong to the peroxidase superfamily [45, 46]. Therefore, the increased activity of these two enzymes may also be related to the biodegradation of BioCNTs, while the precise molecular mechanism of BioCNTs degradation needs further investigation. Meanwhile, the TEM analysis showed that BioCNTs do not translocate into tomato fruits under the tested conditions, alleviating concerns about nanomaterial accumulation in edible tissues. The toxicity evaluation in mice conclusively establish the biocompatibility of the 0.4 mg/L BioCNTs solution and BioCNTs‐treated tomato fruit in mice, with no evidence of systemic toxicity. Therefore, the small size, high carrying efficiency, biocompatibility, and biological safety of BioCNTs make them highly valuable for large‐scale production applications in the future.

Plant viruses can undergo long‐distance systemic movement within host plants [47]. And plant endogenous siRNAs, microRNAs, and mRNAs can move locally between cells through intercellular filaments, as well as over long distances through the vascular system of the phloem [20]. However, exogenously sprayed dsRNA or shRNA can only move locally in plants, which makes long‐distance systematic movement difficult. Direct spraying of dsRNA or shRNA can only result in gene silencing at the spraying site or adjacent areas [10]. Therefore, it is highly important to endow exogenous dsRNA with system mobility to increase the efficacy of exogenous dsRNA‐mediated RNAi and promote the inhibitory effects. TLSs are enriched in the phloem and can be enriched in mRNAs that move at the junction of grafted plants [23]. Based on prior studies and our observations, we propose three non‐exclusive hypotheses. First, the conserved stem‐loop structure of TLS may confer increased structural stability to shRNAs, protecting them from degradation and enabling prolonged systemic accumulation [48]. Second, TLS may act as mobility motifs that facilitate selective loading into the phloem stream, potentially through recognition by RNA‐binding proteins in companion cells [23, 49]. Third, TLS could promote cell‐to‐cell movement of shRNAs by interacting with transport machinery at plasmodesmata or sieve element–companion cell interfaces [20]. While the precise molecular mechanism of TLS systemic movement needs further investigation, our data conclusively demonstrated the ability of TLS to facilitate the systematic movement of exogenous shRNA in *N. benthamiana* and tomato plants. Foliar spraying assays confirmed that TLS can maintain efficacy of shRNA in virus prevention, thereby preventing local virus escape and whole‐plant infection. Therefore, the renovated mobile TLS‐shRNA delivery system can increase the effectiveness of virus prevention and prolong the prevention time, thereby reducing pesticide usage and associated costs. Our findings demonstrate the synergistic potential of TLS‐guided RNA mobility and a BioCNTs delivery system, enabling flexible RNAi molecule design and direct spray‐based application. This integrated approach achieves scalable, non‐transgenic crop protection and confers systemic antiviral effects against both DNA and RNA viruses in plants.

In summary, we developed a mobile and effective antiviral BioCNTs‐based dsRNA/shRNA delivery system that can inhibit both RNA and DNA viruses. Moreover, we revealed that shRNAs can serve as effective exogenous RNAi molecules for the prevention of plant viral diseases. Therefore, this method has promising applications for large‐scale use in fields and greenhouses in the future.

## 4 Experimental Section

### 4.1 Plants Growth Condition and Virus Inoculation

The *Solanum lycopersicum* (variety AC, Moneymaker, and MicroTom) were grown in plots inside growth chambers set at 24/22°C (day/night), 16/8 h (light/dark) photoperiod, and 60% relative humidity (RH). ToMV and TSWV inoculum were prepared, and mechanical inoculation on the second real leaf of tomato seedlings [50], and TYLCV was inoculated with infectious clone on the second real leaf of tomato seedlings [51].

### 4.2 Plasmid Constructions

For dsRNA expression, the dsRNA sequence was shown in Table S12, and cloned into the *Bgl* II and *Kpn* I site of L4440 vector, to produce L4440‐dsRNA^ToMV^, L4440‐dsRNA^TSWV^, and L4440‐dsRNA^TYLCV^. For shRNA expression, the shRNA sequence is shown in Table S12. And cloned into *Xho* I and *Xba* I site of pET30a vector. To produce pET30a‐shRNA^ToMV^, pET30a‐shRNA^TSWV^, and pET30a‐shRNA^TYLCV^ [52].

### 4.3 Synthesis and Extraction of dsRNA and shRNA In Vivo

The above L4440‐dsRNA and pET30a‐shRNA plasmids were transformed into HT115 (DE3). The culture was induced by the addition of isopropyl β‐D‐1 thiogalactopyranoside (IPTG) at a final concentration of 0.5 mm and incubated with shaking at 18°C for 8 h. The culture was centrifuged at 12 000 g for 20 min, and the pellet was resuspended in a 1/40 th volume of TransZol reagent (Transgene, Beijing, ET111‐01) for RNA extraction following the manufacturer's protocols. Dissolve total RNA in RNase‐free water and quantified using NanoDrop 2000 spectrophotometer (Thermo Fisher Scientific).

### 4.4 Synthesis of BioCNTs Nanoparticles

The BioCNTs nanoparticles were synthesized as described previously [19]. The concentration of BioCNTs was measured via absorbance at 632 nm with an extinction coefficient of 0.036 L mg^−1^ cm^−1^. The ultrasonic treatment was performed before use, and added 0.05% Silwet L‐77 in the BioCNTs‐dsRNA/shRNA solution for use in the spraying method.

### 4.5 RNA Loading Assay

To evaluate the loading efficiency of RNA adsorption on BioCNTs, different amounts of BioCNTs were incubated with shRNA cargo (1 µg) for 30 min, respectively. After incubation, all samples were run on agarose gel, and free shRNA (1 µg) was used to assess loading efficiency. For quantitatively evaluated the RNA loading efficiency, 1 µg of each RNA species were incubated with varying amounts of BioCNTs at room temperature for 30 min. The mixtures were then centrifuged at 12 000  rpm for 30 min, and the RNA concentration in the supernatant was measured using a NanoDrop spectrophotometer. The loading efficiency was calculated based on the reduction in free RNA in the supernatant relative to the input amount.

### 4.6 Nuclease Protection Assay

For the nuclease protection assay, free shRNA (1 µg) and 1 µg shRNA on BioCNTs were separately incubated with 100 ng RNase A at room temperature for 2, 5, 10, and 15 min, respectively. After incubation, all shRNA were analyzed through 4% agarose gel with 1 µg shRNA as a reference.

### 4.7 TEM Sample Preparation and Imaging

BioCNTs with different characteristics were imaged using the HITACHI HT7700 transmission electron microscope. A diluted aqueous solution of BioCNTs, BioCNTs‐shRNA^GFP^, or BioCNTs‐dsRNA^GFP^ was dropped onto the plastic wrap and adsorbed onto a copper mesh. To assess BioCNTs distribution and systemic movement, tomato plants were sprayed three times during the growth period with BioCNTs, BioCNTs‐dsRNA^GFP^, or BioCNTs‐TLS‐shRNA^GFP^. Leaves were collected at 24 hpt for cellular localization, while petioles of sprayed leaves, upper stems, and carpopodium were sampled at 28 dpt for translocation analysis. All samples were processed by standard fixation, dehydration, resin embedding, and ultrathin sectioning, followed by TEM imaging on Cu grids.

### 4.8 ROS Accumulation and Enzyme Activity Assays

The accumulation levels of H_2_O_2_ and MDA were measured using the corresponding assay kits (Colorimetric) following the manufacturer's instructions from Solarbio (BC3595, and BC0025, respectively). The enzyme activities of SOD, POD, and CAT were measured using the corresponding assay kits (Colorimetric) following the manufacturer's instructions from Solarbio (BC0170, BC0090, and BC0205, respectively). The samples were collected from H_2_O‐ or BioCNTs‐treated tomato leaves at 2, 4, and 6 dpt. Each biological replicate involved pooled samples from 4 individual plants, and a total of 3 biological replicates were conducted.

### 4.9 Measurement of Chlorophyll Fluorescence

Chlorophyll fluorescence was assessed in vivo using fully expanded leaves of tomato plants. The LI‐6400XT photosynthesis system from Li‐Cor Biosciences (Lincoln, NE) equipped with a leaf chamber fluorometer (Li‐Cor Part No.6400‐40, enclosed leaf area: 2 cm^2^) was utilized, following the manufacturer's instructions. The measurements were taken at a leaf temperature of ∼22°C, with the light source consisting of a mixture of blue (10%) and red (90%) LEDs.

### 4.10 BioCNTs Treatment Assay

The BioCNTs were co‐incubated with dsRNA, shRNA, or TLS‐shRNA, respectively, and diluted with sterile double distilled water (ddH_2_O) with 0.05% Silwet L‐77 immediately before use. The BioCNTs solution or ddH_2_O with 0.05% Silwet L‐77 was sprayed onto tomato or *N. benthamiana* leaves.

For the virus control effect of BioCNTs‐shRNA/dsRNA, the BioCNTs, and BioCNTs‐shRNA/dsRNA were sprayed on the tomato leaves. At 1 dpt, the sprayed first true leaf was inoculated with ToMV, TSWV, or TYLCV. The BioCNTs, BioCNTs‐shRNA, and BioCNTs‐dsRNA‐treated upper leaf was collected at 30 dpi for further analysis.

For the virus control effect of BioCNTs‐TLS‐shRNA, the tomato plants were first inoculated with ToMV or TYLCV. At 14 dpi, the BioCNTs‐TLS‐shRNA were sprayed on the tomato leaves. At 7 and 14 dpt, the upper‐untreated leaves were collected for further analysis.

For the co‐infection of ToMV and TYLCV, the tomato seedlings were pre‐inoculated with TYLCV, and 14 days after TYLCV inoculation, the plants were inoculated with ToMV. Then, at 7 days after ToMV inoculation, the BioCNTs, TLS‐shRNA^To+TY^, and BioCNTs‐TLS‐shRNA^To+TY^ were sprayed. At 14 dpt, the upper‐untreated leaves were collected for further analysis.

### 4.11 RNA Isolation and Quantification

The total RNA was extracted using TransZol reagent (Transgene, Beijing, ET111‐01). First strand cDNA was synthesized using total RNA (2 µg) per reaction (20 µL) following the manufacturer's protocols (Aidlab, Beijing, PC7002). RT‐PCR was performed using Taq Master Mix (Dye Plus), and qPCR was performed using a FastSYBR reaction kit as instructed (CWBIO, Beijing, CW2621M). To quantity target shRNAs, we performed stem‐loop RT‐qPCR as described [53]. Primers were designed using NCBI Primer‐BLAST software and are listed in Table S12. The qPCR data were analyzed using the 2^−ΔΔCT^ method [54]. Statistical differences between treatments were determined using the one‐way ANOVA followed by the Tukey's multiple test. All experiments were performed with at least three biological replicates per treatment with similar results.

### 4.12 RNA‐seq and Bioinformation Analysis

Leaf samples were collected from tomato seedlings at 4 days after BioCNTs treatment or H_2_O‐control treatment and 14, 28 days after BioCNTs‐TLS‐shRNA, TLS‐shRNA, BioCNTs, or H_2_O control treatment. Then, the total RNA was extracted as described above, and RNA integrity was assessed with a NanoDrop spectrophotometer and Agilent 5300 Bioanalyzer. cDNA libraries were constructed using the Illumina Stranded mRNA Prep, Ligation (San Diego, CA), and paired‐end sequencing (300 bp) was performed on the Illumina NovaSeq X Plus platform by Shanghai Majorbio Bio‐pharm Biotechnology (China). Raw reads were quality‐filtered using fastp (v0.23.4), and clean reads were mapped to the *Solanum lycopersicum* reference genome SL3.0 (https://data.jgi.doe.gov/refine‐download/phytozome?organism=Slycopersicum&expanded=514) using HISAT2 (v2.2.1). Gene‐level read counts were obtained using featureCounts (v2.1.1), and differential expression analysis was conducted with DESeq2 (v1.42.0) in R. Genes with |log_2_ (fold change)| ≥ 1 and adjusted *p*‐value < 0.05 were considered significantly differentially expressed. Gene Ontology (GO) and KEGG pathway enrichment analyses were performed using the Goatools and Python scipy software to identify biological processes and pathways affected by BioCNTs treatment.

### 4.13 Confocal Microscopy

For the observation of GFP fluorescence intensity in 16C *N. benthamiana*, the leaf samples were analyzed with a confocal laser scanning microscope (Leica STELLARIS 5, Germany). For the GFP, the excitation wavelength was set at 488 nm and the emission wavelength at 510–550 nm with a 10% intensity and a 580 gain. The Image J software was used for fluorescence intensity statistics [55]. For each image, 3‐5 regions of interest (ROIs) were manually selected within GFP‐expressing mesophyll cells. The integrated density (total pixel intensity) and mean intensity were recorded. Background fluorescence was measured from adjacent non‐GFP areas and subtracted from each ROI measurement. The resulting values were averaged across replicates for statistical analysis.

### 4.14 In Situ Hybridization

The plant samples were fixed in FAA fixative (3.7% formaldehyde, 0.5% acetic acid, 50% ethanol) overnight and embedded in paraffin. Histological sections were prepared and incubated with probes using a DIG in situ hybridization kit (BersinBio, Guangzhou, China). The DIG‐labeled probes were designed and synthesized by Sangon Biotechnology Co., Ltd. (Shanghai, China).

### 4.15 Concentration of Lycopene and Degrees Brix

The lycopene concentration was tested using Plant lycopene ELISA kit (CB11299‐Pt, Coibo Bio). The degrees Brix was determined using a digital refractometer (PAL‐1, ATAGO), adjusted and calibrated at 20°C with distilled water. More than six red ripe fruits were collected from each treatment.

### 4.16 Toxicity Evaluation

The male Kunming (KM) mice (6‐8 weeks old) were provided by SPF (Suzhou) Biotechnology Co., Ltd (Suzhou, China). Mice were maintained in the animal facility in Dr. Can Biotechnology (Zhejiang) Co., Ltd, under standard conditions with free access to food and water. After adaptation for 1 week, the mice were randomly distributed into 4 groups (5 mice for each group): saline control, tomato fruit from untreated plants, tomato fruit from BioCNTs‐treated plants, and BioCNTs solution (0.4 mg/L, the working concentration). Each mouse received 100 µL of the respective sample via oral gavage daily for 7 consecutive days. Four hours after the final administration, blood and organs were collected for analysis. All experiments in this research were complied with the principles and guidelines for the use of laboratory animals and approved by the Lab of Animal Experimental Ethical Inspection of Dr. Can Biotechnology (Zhejiang) Co., Ltd (No. DRK‐20250901001).

## Author Contributions

X.D.L. and S.J.L. designed the experiments. X.D.L., X.F.L., Z.P.C., Z.L., and S.J.L. performed most experiments. X.D.L., X.F.L., Z.P.C., Z.L., X.F.W., C.Y.Z., M.J.C., and S.J.L. analyzed data. X.D.L., M.J.C., and S.J.L. wrote the manuscript. All authors agreed with the results and discussions presented in the manuscript.

## Conflicts of Interest

The authors declare no conflicts of interest.

## Supporting information




**Supporting File 1**: advs73479‐sup‐0001‐SuppMat.pdf.

**Supporting File 2**: advs73479‐sup‐0002‐Supplemental‐Tables.xlsx.

## Data Availability

The data that support the findings of this study are available in the supplementary material of this article.
