# Supplementary material for: BioCNTs Mediated Delivery of Systemically Mobile Small RNAs via Leaf Spray to Control Both Tomato DNA and RNA Viruses
Source: Adv Sci (Weinh). 2025 Dec 21;13(10):e04889. doi: 10.1002/advs.202504889 (PMC12915072; doi:10.1002/advs.202504889)
Supplement: Supplementary file 1 — Supporting File 1: advs73479‐sup‐0001‐SuppMat.pdf. [file ADVS-13-e04889-s001.pdf]

Supporting Information

BioCNTs mediated delivery of systemically mobile small RNAs via leaf spray to control both tomato DNA and RNA viruses

Xuedong Liu[1,3], Xiaofei Liang[1], Zipeng Cai[2], Zheng Liu[1], Xuefeng Wang[1], Changyong Zhou[1], Mengji Cao[1], Sijia Liu[2]**

This PDF file includes:

Figs. S1-S21

Figure S1

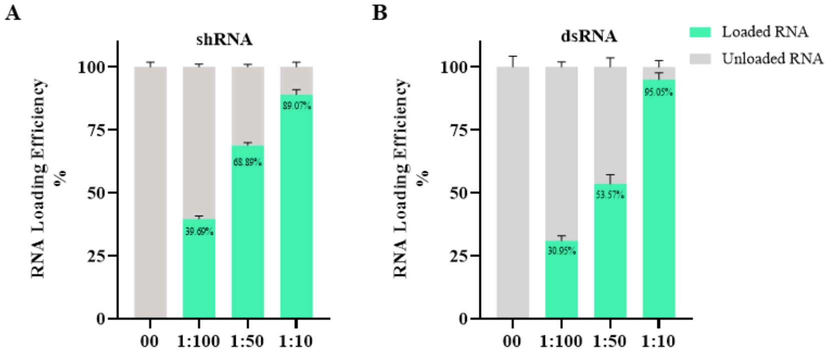

Figure S1. Evaluation of the RNA loading efficiency of BioCNTs. A) The loading efficiency of BioCNTs on shRNA. B) The loading efficiency of BioCNTs on dsRNA.

Figure S2

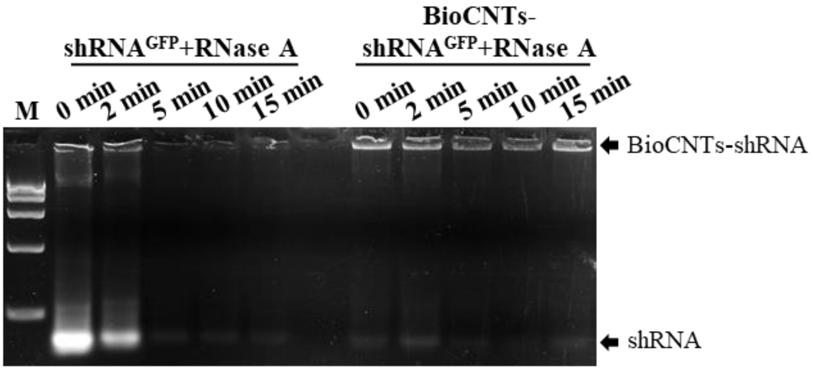

Figure S2. The shRNA was protected by BioCNTs. The left panel stand for free shRNA^{GFP} with RNase A treatment for 0, 2, 5, 10, or 15 min at room temperature; the right panel stand shRNA^{GFP} loaded onto BioCNTs and treated with RNase A for 0, 2, 5, 10, or 15 min at room temperature.

Figure S3

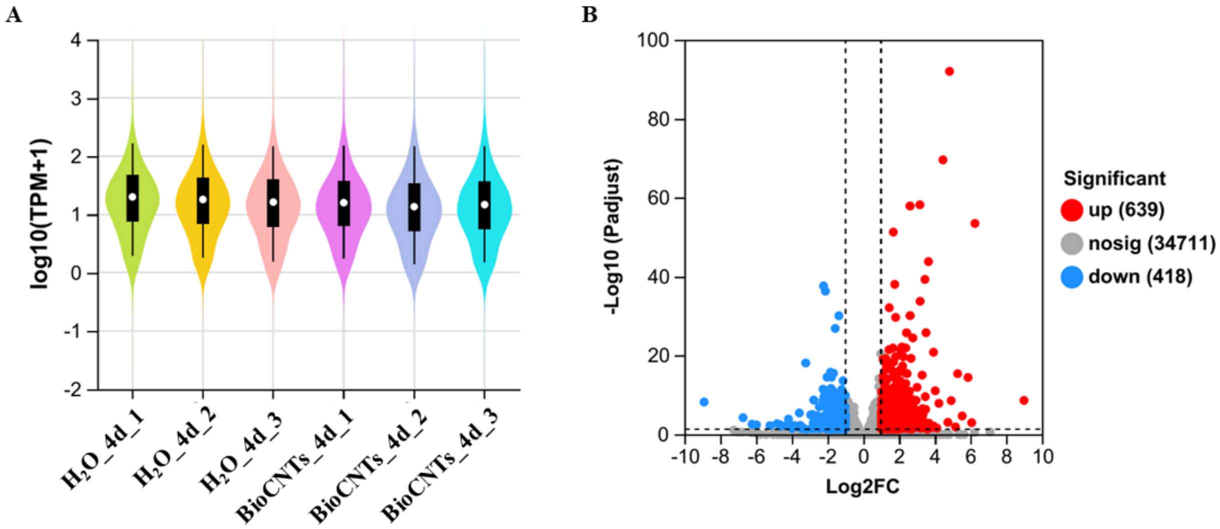

Figure S3. Transcriptome gene expression statistical analysis after H_2O and BioCNTs treatment for 4 days. A) The distribution of genes expression levels of H_2O and BioCNTs treatment tomato plants. B) The different expression genes (DEGs) under BioCNTs treatment. Each point represents a specific gene. The red points indicating significantly upregulated genes, blue points indicating significantly downregulated genes, and gray points indicating non significantly differentially expressed genes.

Figure S4

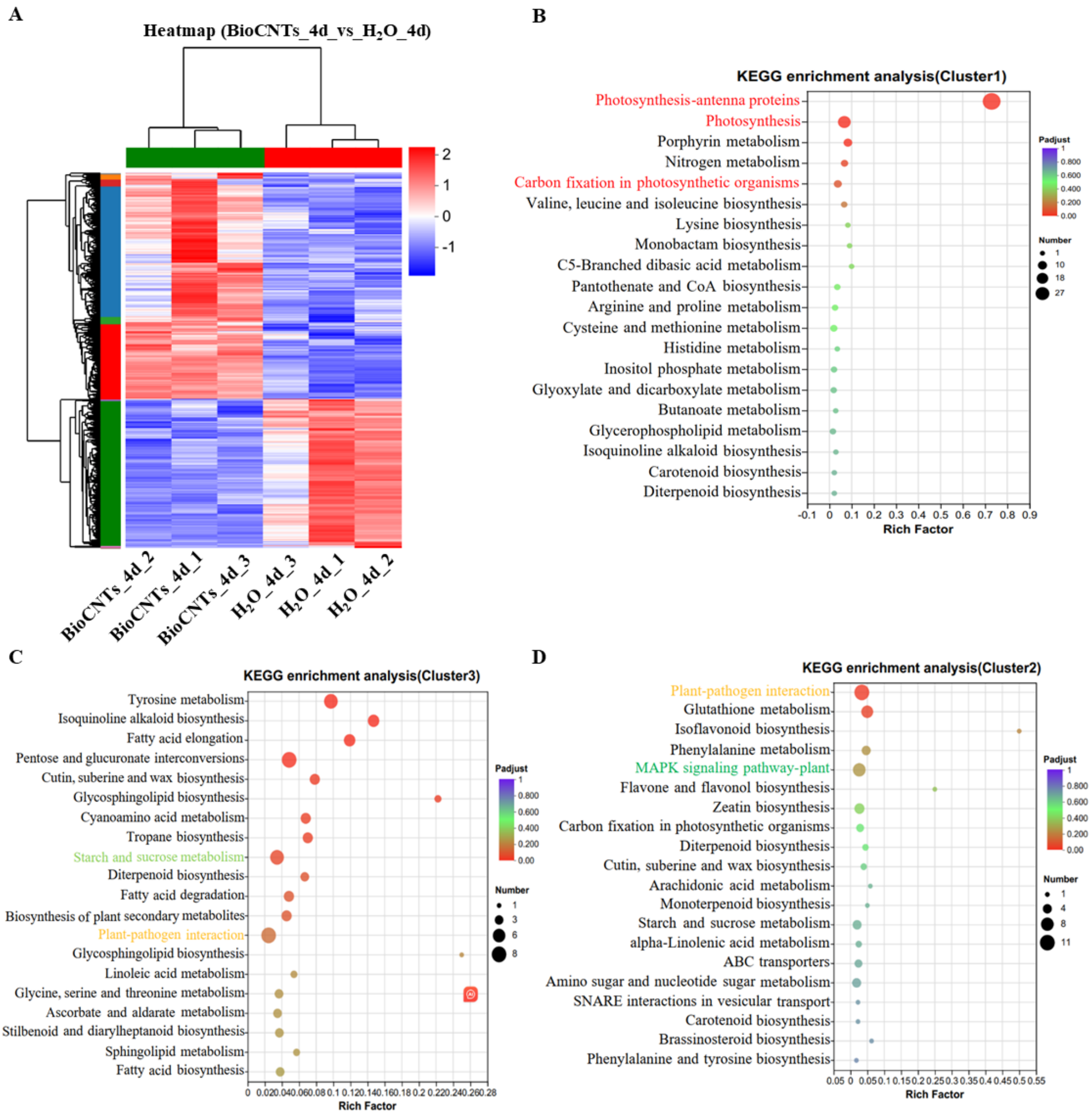

Figure S4. Main regulatory pathways involved in tomato leaves response to BioCNTs treatment. A) Heatmap of DEGs in tomato leaves at 4 days post BioCNTs treatment (dpt). B, C, D) KEGG analysis of the genes in top three clusters.

Figure S5

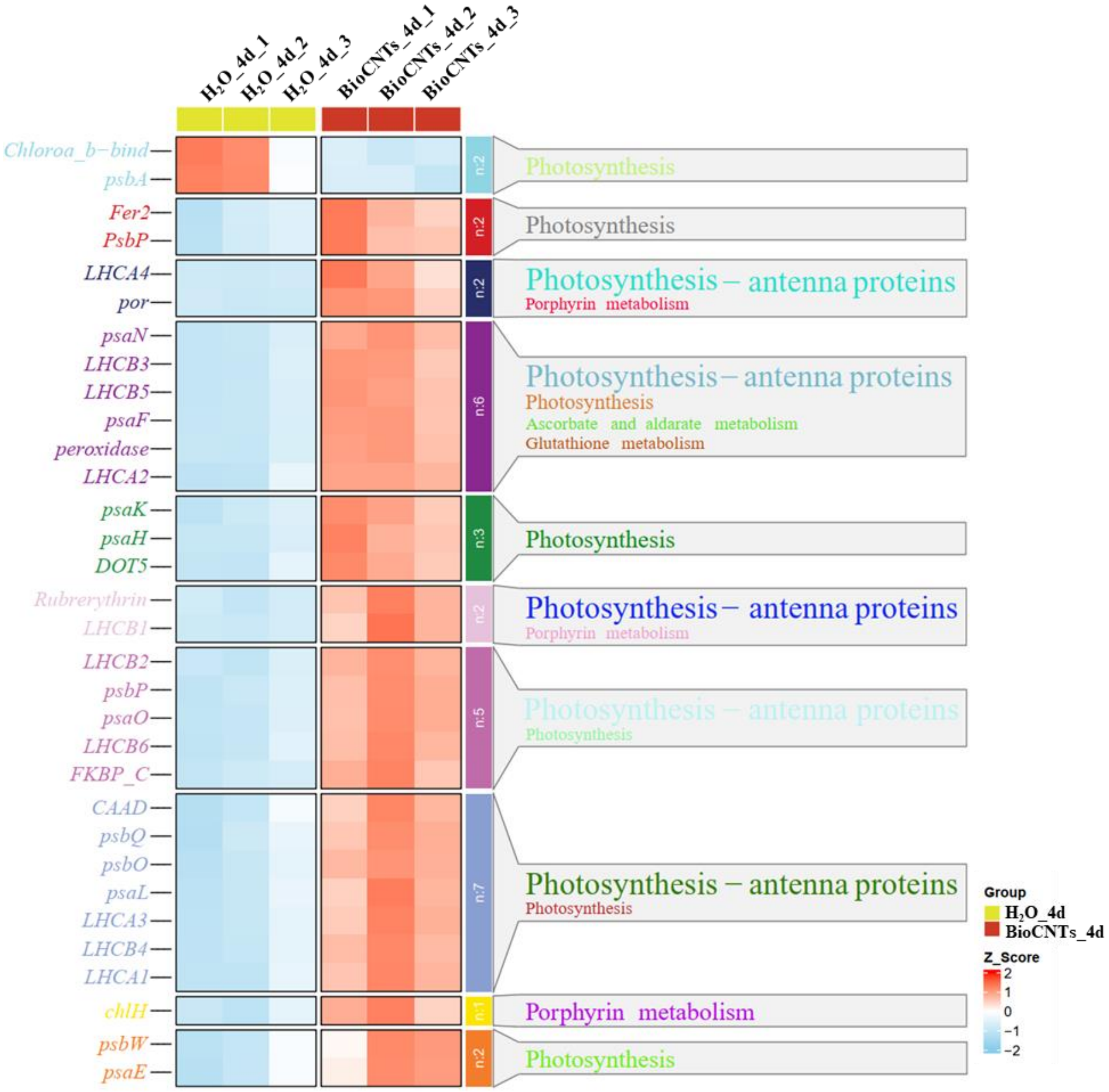

Figure S5. The combined analysis of clustering and pathway enrichment of photosynthesis-related genes after BioCNTs treatment for 4 days. Most photosynthesis pathway-related genes were up-regulated in BioCNTs-treated tomato plants.

Figure S6

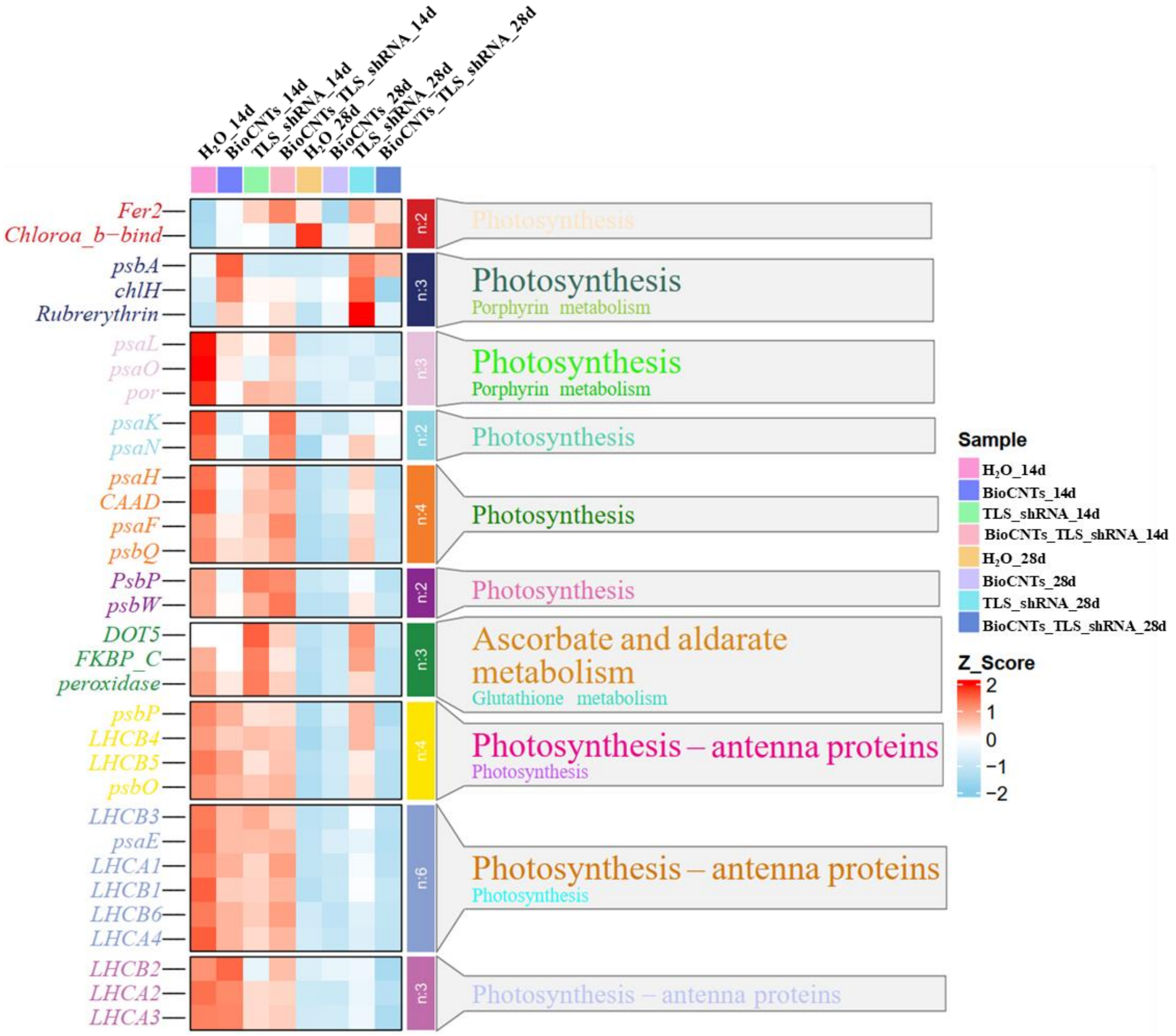

Figure S6. The combined analysis of clustering and pathway enrichment of photosynthesis-related genes following treatment with BioCNTs, TLS-shRNA^{GFP}, and BioCNTs-TLS-shRNA^{GFP} for 14 days and 28 days.

Figure S7

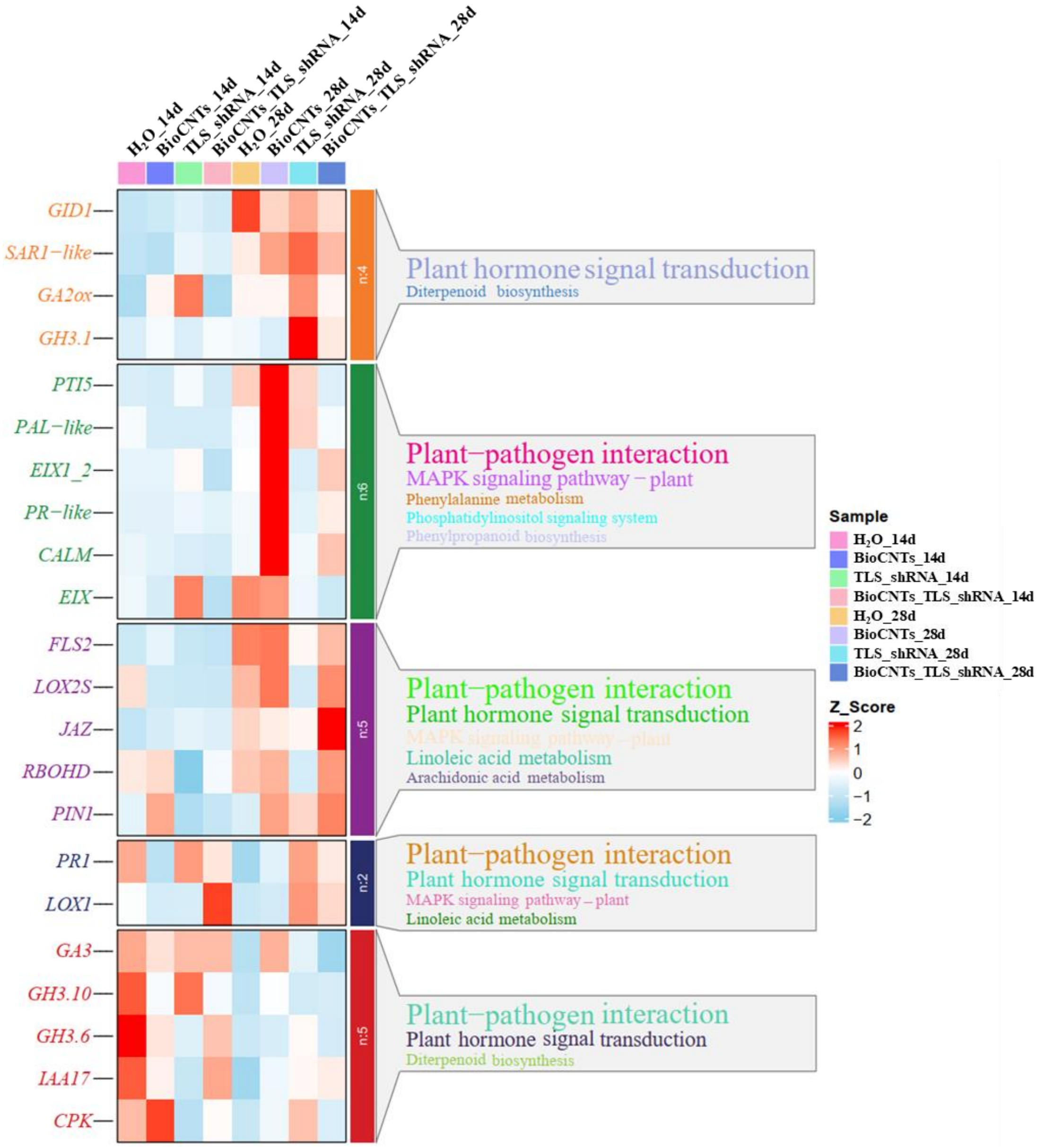

Figure S7. The combined analysis of clustering and pathway enrichment of plant resistance pathway after BioCNTs, TLS-shRNA^{GFP}, and BioCNTs-TLS-shRNA^{GFP} treatment for 14 days and 28 days.

Figure S8

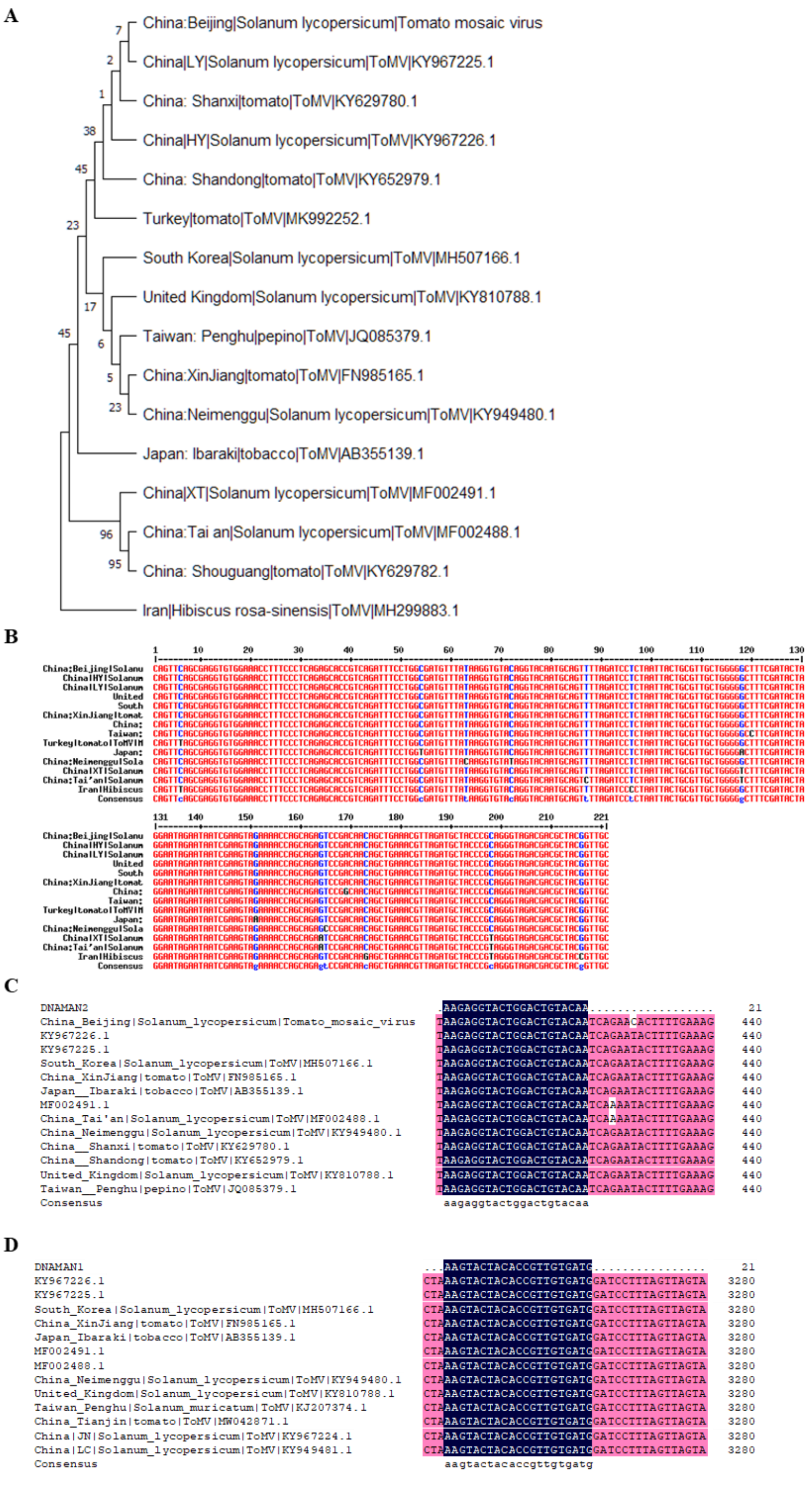

Figure S8. Homology analysis of ToMV CP and replication protein. A) The phylogenetic tree for different isolation of ToMV CP. Maximum likelihood analysis with 1,000 replicates was performed using the Mega 6 software; B) Multiple amino acid sequence alignment was done using the software at http://www.clustal.org/clustal2/; C) Conservative alignment analysis of ToMV CP target sequence through DNAMAN software; D) Conservative alignment analysis of ToMV replication protein target sequence through DNAMAN software.

Figure S9

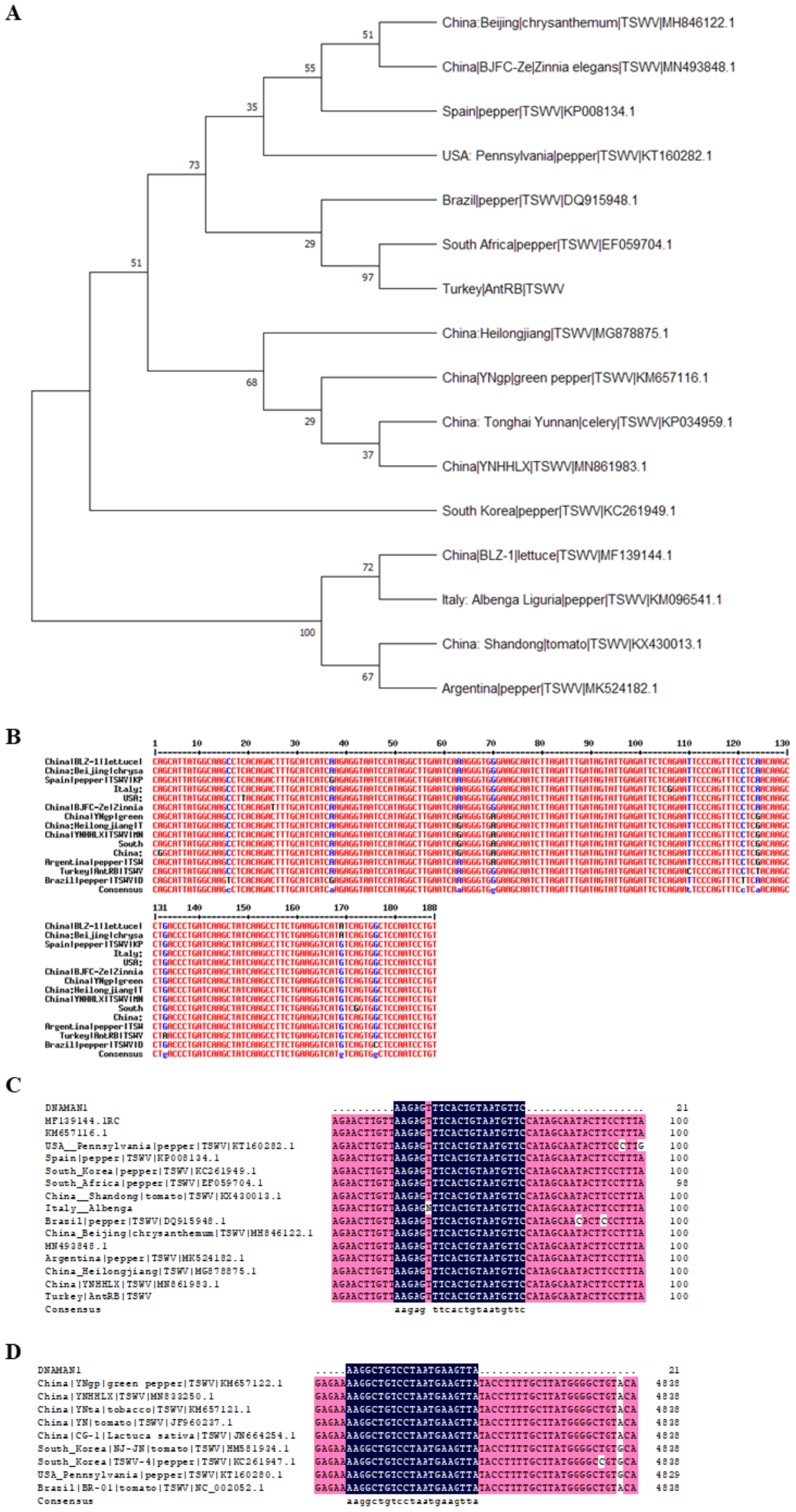

Figure S9. Homology analysis of TSWV CP and replication protein. A) The phylogenetic tree for different isolation of TSWV CP. Maximum likelihood analysis with 1,000 replicates was performed using the Mega 6 software; B) Multiple amino acid sequence alignment was done using the software at http://www.clustal.org/clustal2/; C) Conservative alignment analysis of TSWV CP target sequence through DNAMAN software; D) Conservative alignment analysis of TSWV replication protein target sequence through DNAMAN software.

Figure S10

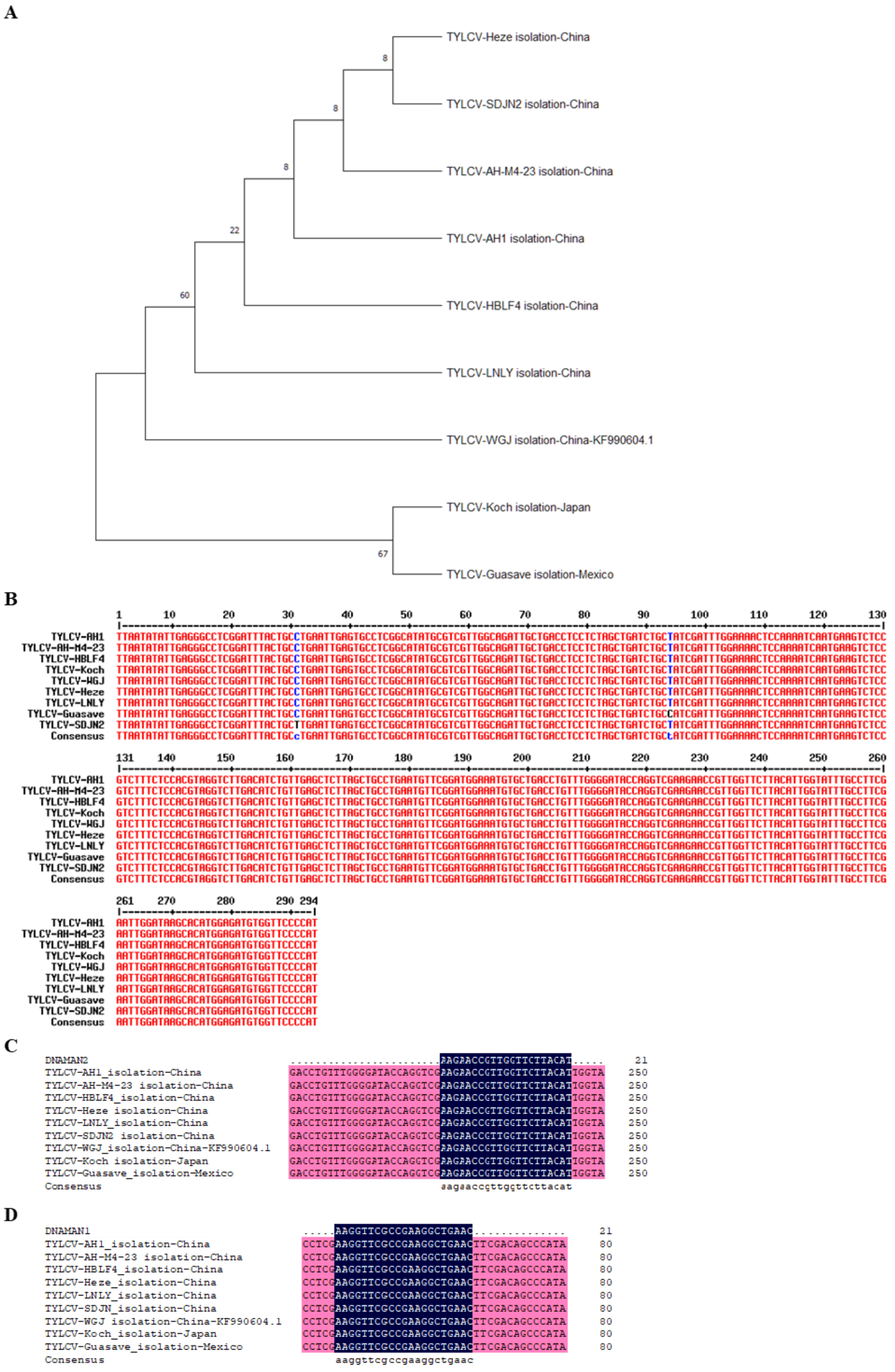

Figure S10. Homology analysis of TYLCV AC4 and AV1. A) The phylogenetic tree for different isolation of TYLCV AC4. Maximum likelihood analysis with 1,000 replicates was performed using the Mega 6 software; B) Multiple amino acid sequence alignment was done using the software at http://www.clustal.org/clustal2/; C) Conservative alignment analysis of TYLCV AC4 target sequence through DNAMAN software; D) Conservative alignment analysis of TYLCV AV1 target sequence through DNAMAN software.

Figure S11

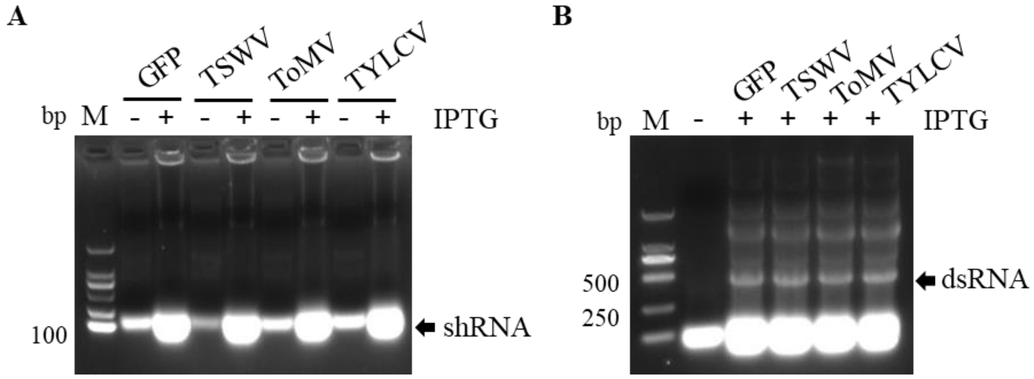

Figure S11. The detection of induced-target shRNA (A) and dsRNA (B) in HT115 (DE3) system through agarose gel electrophoresis.

Figure S12

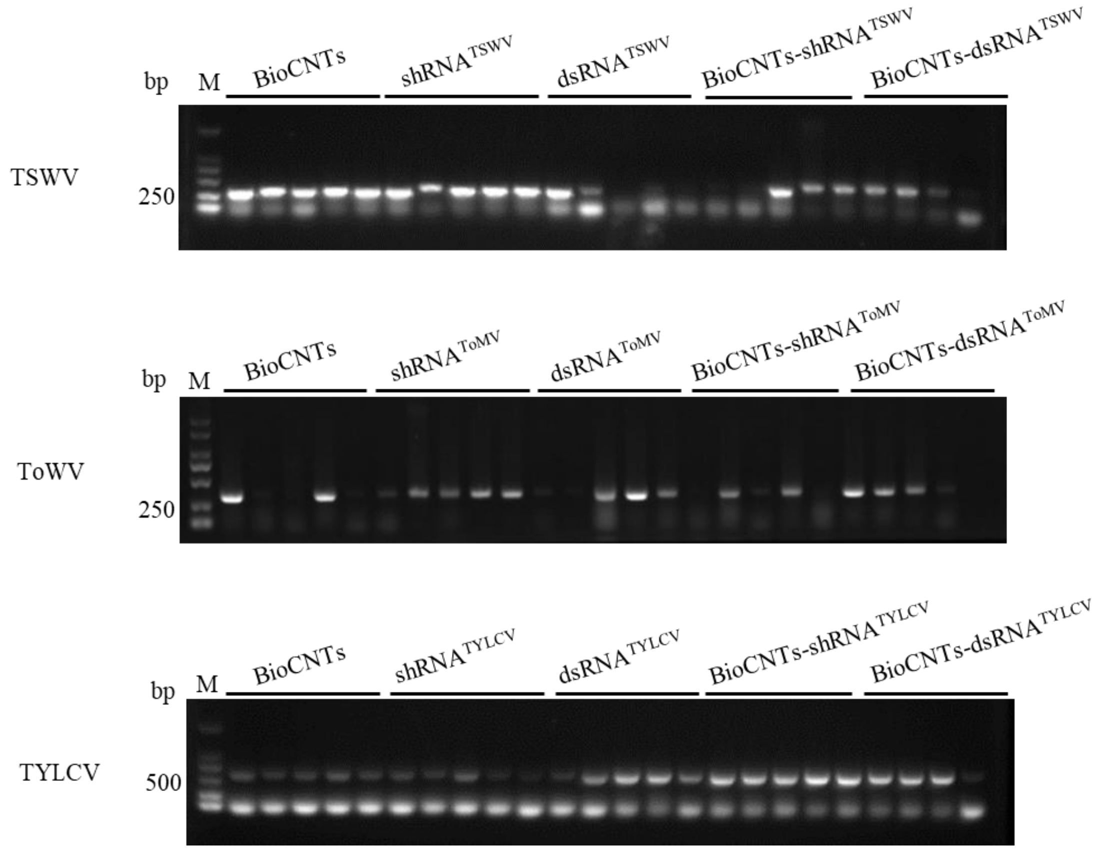

Figure S12. PCR detection of TSWV (A), ToMV (B), TYLCV (C) infection incidence.

Figure S13

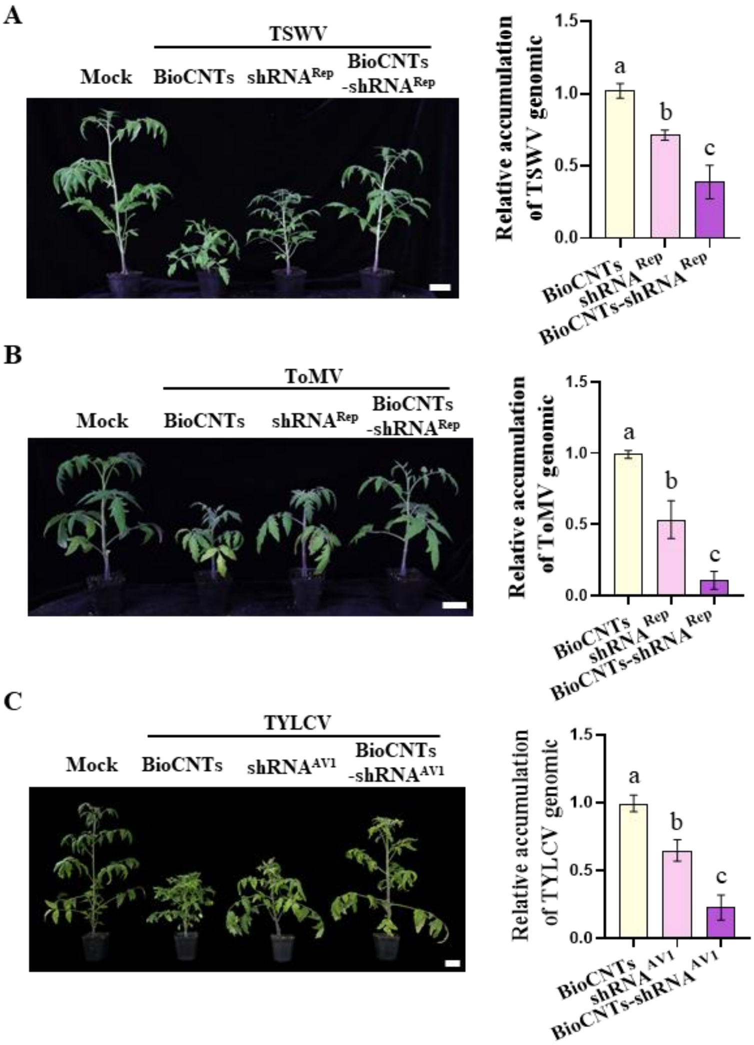

Figure S13. The BioCNTs-shRNA system targeting the ToMV/TSWV replication protein or TYLCV AV1 also significantly inhibits viral infection. A) The BioCNTs-shRNA^{Rep}-treatment inhibited TSWV infection with milder symptoms, and RT-qPCR results showed significantly decreased TSWV RNA accumulation in tomato plants (BioCNTs-shRNA^{Rep}) compared with that in the control (BioCNTs, shRNA^{Rep}-treatment) plants. B) The BioCNTs-shRNA^{Rep}-treatment inhibited ToMV infection with milder symptoms, and RT-qPCR results showed significantly decreased ToMV RNA accumulation in tomato plants (BioCNTs-shRNA^{Rep}) compared with that in the control (BioCNTs, shRNA^{Rep}-treatment) plants. C) The BioCNTs-shRNA^{AV1}-treatment inhibited TYLCV infection with milder symptoms, and RT-qPCR results showed significantly decreased TYLCV DNA accumulation in tomato plants (BioCNTs-shRNA^{AV1}) compared with that in the control (BioCNTs, shRNA^{AV1}-treatment) plants. Scale bars = 5 cm (A-C). The different letters above each bar in A-C indicated statistically significant differences as determined by a one-way ANOVA followed by Tukey's multiple test ($p < 0.05$), error bars were SEM. These experiments were performed three times and had at least six biological replicates per treatment.

Figure S14

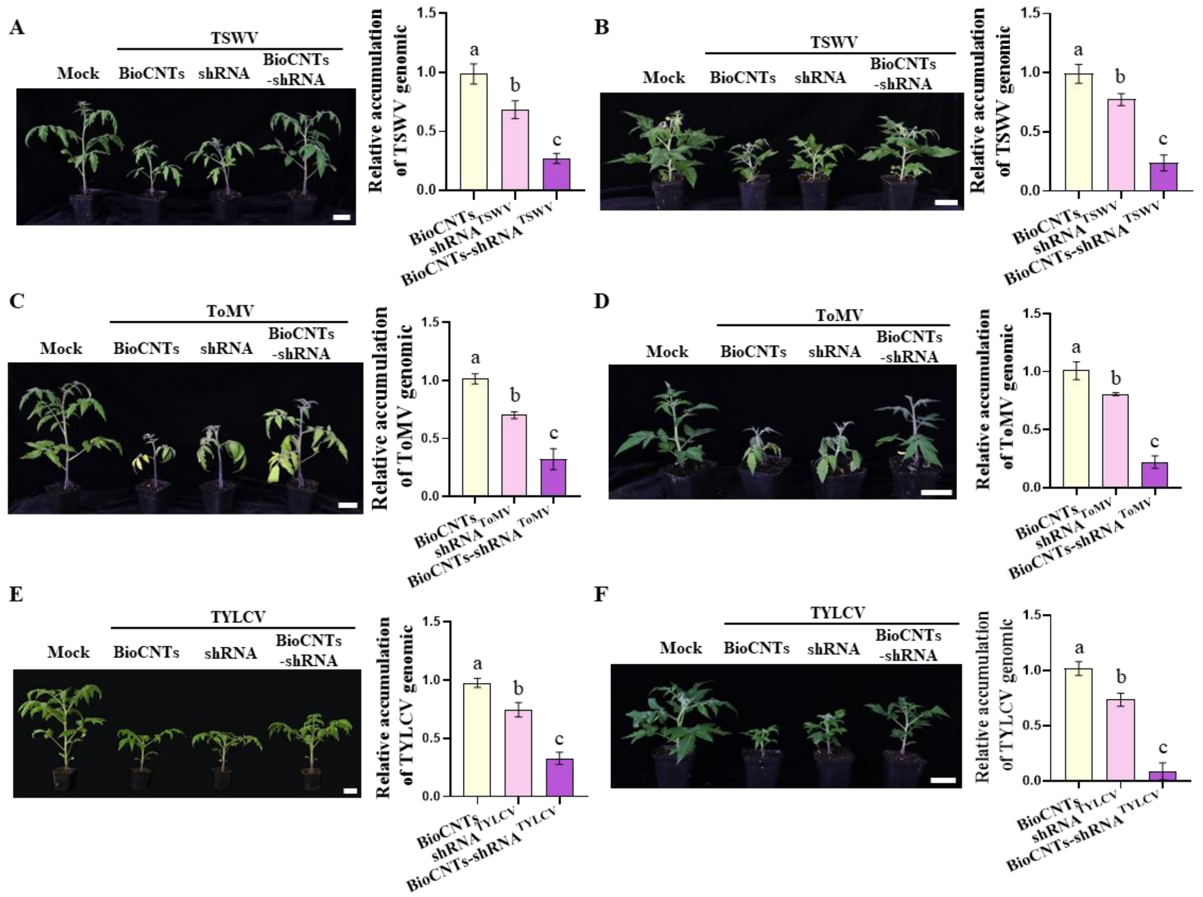

Figure S14. BioCNTs-shRNA has a wide range of virus protection capabilities across different tomato varieties. A, B) The BioCNTs-shRNA^{TSWV}-treatment inhibited TSWV infection with milder symptoms in Moneymaker (A) and MicroTom (B) varieties. The RT-qPCR results showed significantly decreased TSWV RNA accumulation in Moneymaker and MicroTom tomato plants (BioCNTs-shRNA^{TSWV}) compared with that in the control (BioCNTs, or shRNA^{TSWV}-treatment) plants. C, D) The BioCNTs-shRNA^{ToMV}-treatment inhibited ToMV infection with milder symptoms in Moneymaker (C) and MicroTom (D) varieties. The RT-qPCR results showed significantly decreased ToWV RNA accumulation in tomato plants (BioCNTs-shRNA^{ToMV}) compared with that in the control (BioCNTs, or shRNA^{ToMV}-treatment) plants. E, F) The BioCNTs-shRNA^{TYLCV}-treatment inhibited TYLCV infection with milder symptoms in Moneymaker (E) and MicroTom (F) varieties. The RT-qPCR results showed significantly decreased TYLCV DNA accumulation in tomato plants (BioCNTs-shRNA^{TYLCV}) compared with that in the control (BioCNTs, or shRNA^{TYLCV}-treatment) plants. Scale bars = 5 cm (A-F). The different letters above each bar in A-F indicated statistically significant differences as determined by a one-way ANOVA followed by Tukey's multiple test ($p < 0.05$), error bars were SEM. These experiments were performed three times and had at least six biological replicates per treatment.

Figure S15

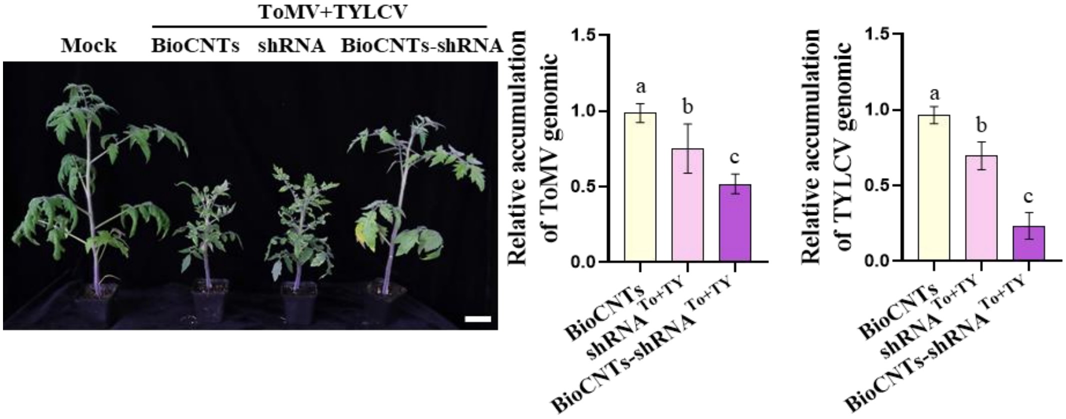

Figure S15. BioCNTs exhibited synergistic antiviral efficacy in co-infected tomato plants. BioCNTs-shRNA^{To+TY} inhibited both ToMV and TYLCV infections, resulting in milder symptoms. RT-qPCR analysis revealed significantly reduced accumulation of ToMV RNA and TYLCV DNA in BioCNTs-shRNA^{To+TY}-treated plants compared with control plants treated with either BioCNTs or naked shRNA^{To+TY}. Scale bar = 5 cm. The different letters above each bar indicated statistically significant differences as determined by a one-way ANOVA followed by Tukey's multiple test ($p < 0.05$), error bars were SEM. These experiments were performed three times and had at least six biological replicates per treatment.

Figure S16

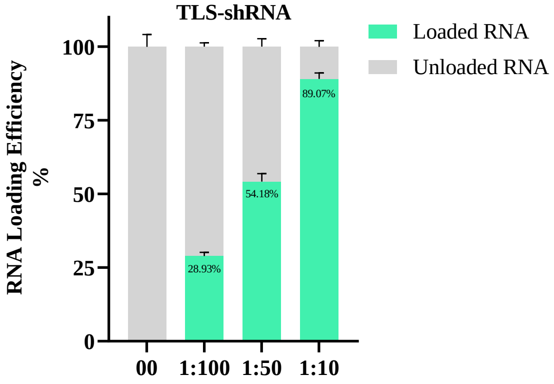

Figure S16. The TLS-shRNA loading efficiency of BioCNTs.

Figure S17

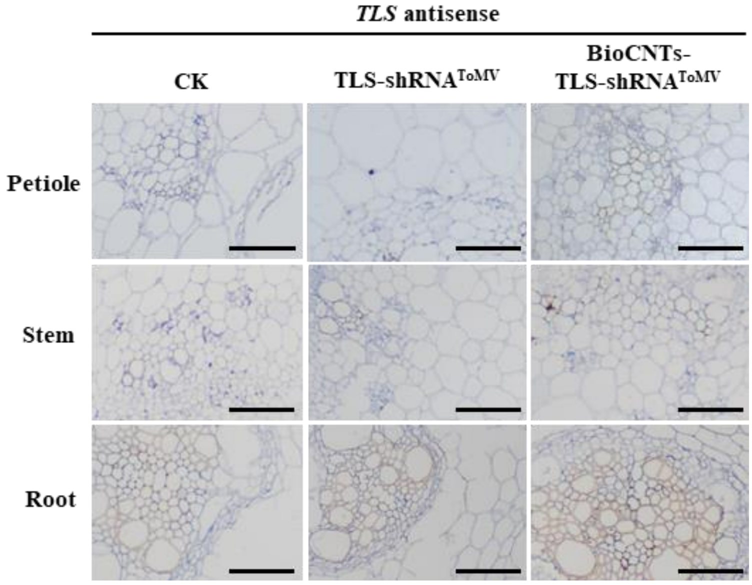

Figure S17. RNA *in situ* hybridization with DIG-labeled probes to *TLS*. The roots, stems, and petioles tissues of upper untreated leaf were collected from BioCNTs, TLS-shRNA^{ToMV}, and BioCNTs-TLS-shRNA^{ToMV}-treated tomato plants. Scale bars = 15 μm.

Figure S18

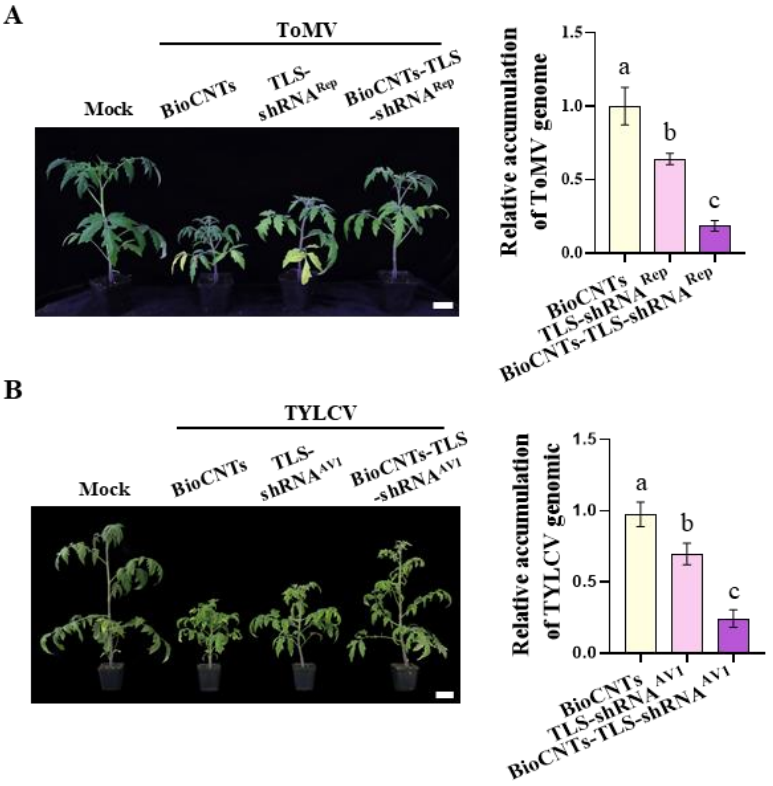

Figure 18. The BioCNTs-TLS-shRNA system targeting ToMV replication protein or TYLCV AV1 significantly inhibits viral infection. A) The BioCNTs-TLS-shRNA^{Rep}-treatment inhibited ToMV infection with milder symptoms, and RT-qPCR results showed significantly decreased ToMV RNA accumulation in tomato plants (BioCNTs-TLS-shRNA^{Rep}) compared with that in the control (BioCNTs, TLS-shRNA^{Rep}-treatment) plants. B) The BioCNTs-TLS-shRNA^{AV1}-treatment inhibited TYLCV infection with milder symptoms, and RT-qPCR results showed significantly decreased TYLCV DNA accumulation in tomato plants (BioCNTs-TLS-shRNA^{AV1}) compared with that in the control (BioCNTs, TLS-shRNA^{AV1}-treatment) plants. Scale bars = 5 cm (A, B). The different letters above each bar in A and B indicated statistically significant differences as determined by a one-way ANOVA followed by Tukey's multiple test ($p < 0.05$), error bars were SEM. These experiments were performed three times and had at least six biological replicates per treatment.

Figure S19

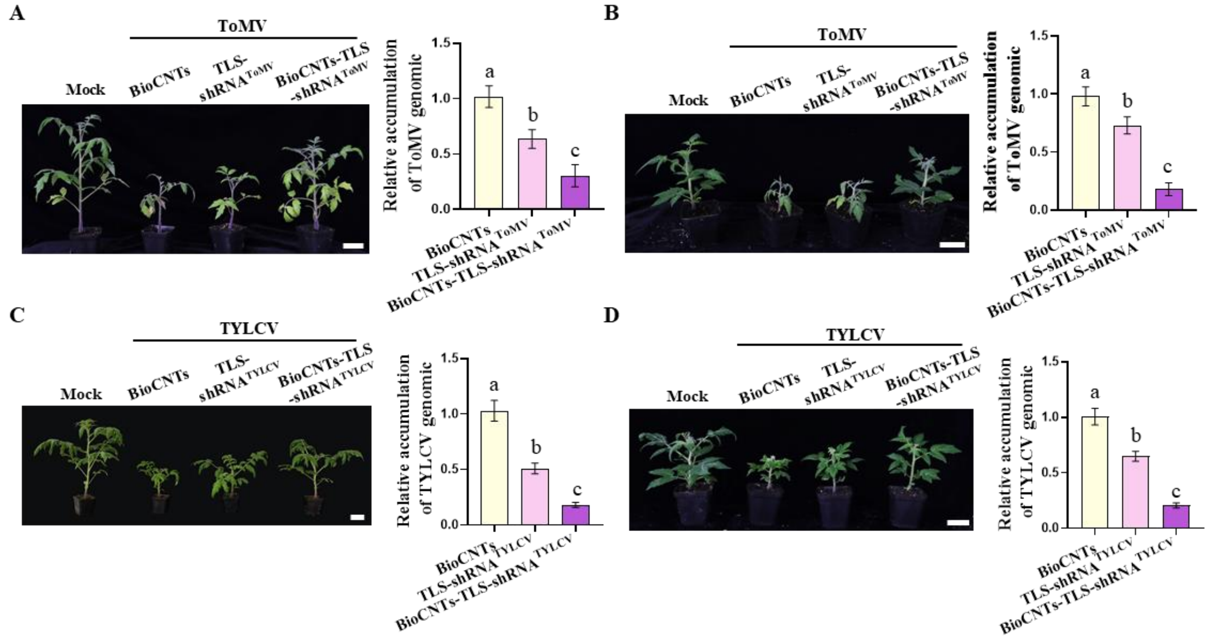

Figure S19. BioCNTs-TLS-shRNA has a wide range of virus protection capabilities across different tomato varieties. A, B) The BioCNTs-TLS-shRNA^{ToMV}-treatment inhibited ToMV infection with milder symptoms in Moneymaker (A) and MicroTom (B) varieties. The RT-qPCR results showed significantly decreased ToWV RNA accumulation in tomato plants (BioCNTs-TLS-shRNA^{ToMV}) compared with that in the control (BioCNTs, or TLS-shRNA^{ToMV}-treatment) plants. C, D) The BioCNTs-TLS-shRNA^{TYLCV}-treatment inhibited TYLCV infection with milder symptoms in Moneymaker (C) and MicroTom (D) varieties. The RT-qPCR results showed significantly decreased TYLCV DNA accumulation in tomato plants (BioCNTs-TLS-shRNA^{TYLCV}) compared with that in the control (BioCNTs, or TLS-shRNA^{TYLCV}-treatment) plants. Scale bars = 5 cm (A-D). The different letters above each bar in A-F indicated statistically significant differences as determined by a one-way ANOVA followed by Tukey's multiple test ($p < 0.05$), error bars were SEM. These experiments were performed three times and had at least six biological replicates per treatment.

Figure S20

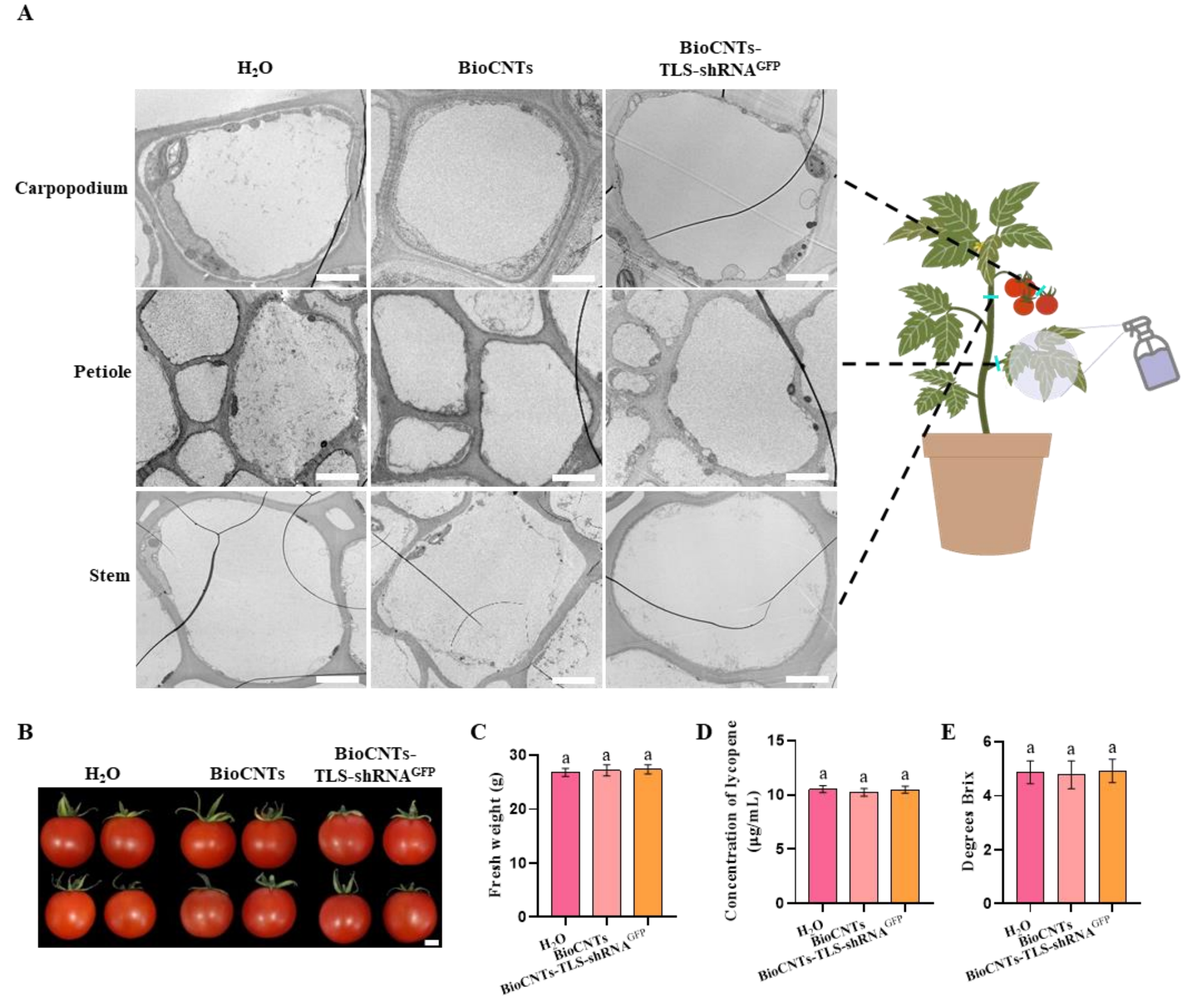

Figure S20. BioCNTs remain localized and do not translocate to or affect the tomato fruit. A) Observation the presence of BioCNTs and BioCNTs-TLS-shRNA^{GFP} in vascular bundles. The BioCNTs are not observed in the carpopodium (the upper panel), the petiole of treatment leaves (the middle panel), and the upper stem (the lower panel). Scale bars = 2 μm. B) Photographs of fresh fruits from H_2O (control), BioCNTs, and BioCNTs-TLS-shRNA^{GFP}-treated plants. Scale bar = 1 cm. C-E) Fresh weight (C), lycopene concentration (D), and degrees Brix (E) of the fruits under BioCNTs and BioCNTs-TLS-shRNA^{GFP} treatment. The different letters above each bar in C-E indicated statistically significant differences as determined by a one-way ANOVA followed by Tukey's multiple test ($p < 0.05$), error bars were SEM. These experiments were performed two times and more than six red ripe fruits were collected from each treatment.

Figure S21

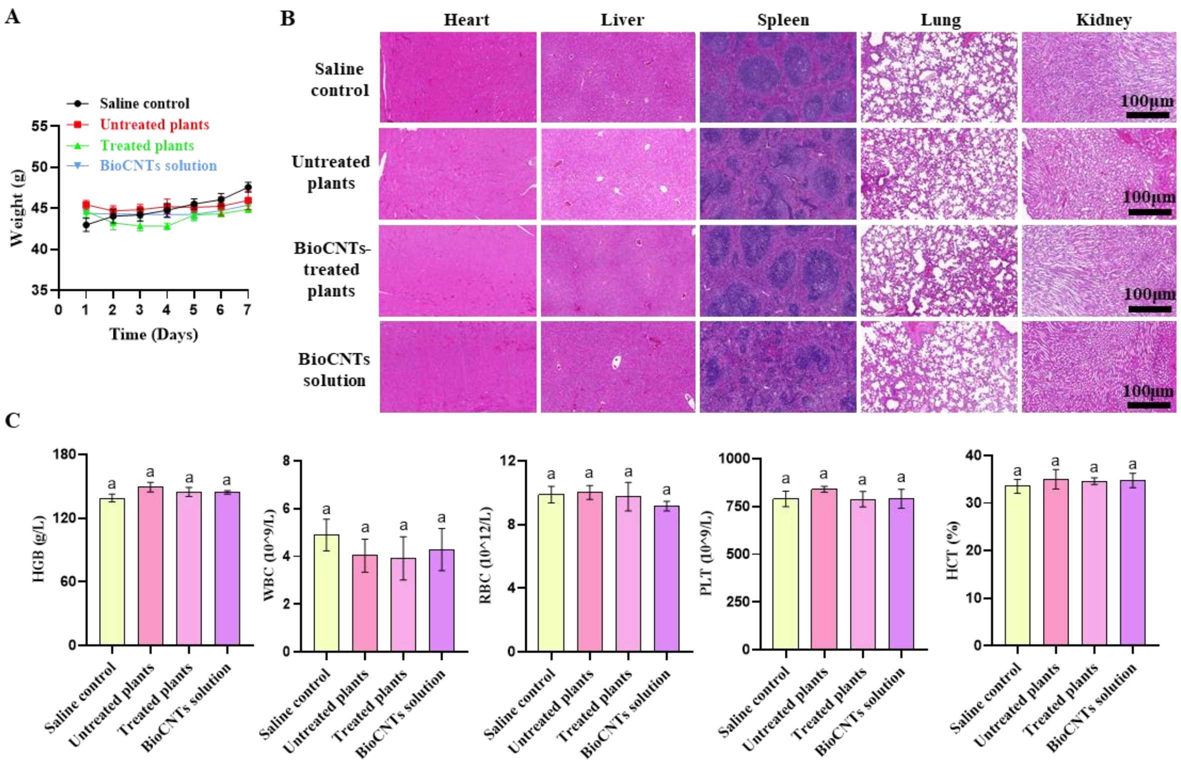

Figure S21. BioCNTs treatment exhibits biosafety in mice. A) The weight of mice feeding with saline (Saline control), tomato fruit from untreated plants (Untreated plants), tomato from BioCNTs-treated plants (Treated plants), and BioCNT solution. B) The histopathological examination of heart, liver, spleen, lung and kidney tissues in mice from feeding with saline, tomato fruit from untreated plants, tomato from BioCNTs-treated plants, and BioCNT solution (0.4 mg/L). Scale bars = 100 μm. C) The effects of BioCNTs on hematological profile. The changes of HGB (hemoglobin), WBC (white blood cell), RBC (red blood cell), PLT (platelet), HCT (hematocrit) in mice from above treatment. The different letters above each bar indicated statistically significant differences as determined by a one-way ANOVA followed by Tukey's multiple test ($p < 0.05$), error bars were SEM, n = 5 per group.
